# Novel diagnostic biomarkers of oxidative stress, immune- infiltration characteristics and experimental validation of SERPINE1 in colon cancer

**DOI:** 10.1007/s12672-023-00833-w

**Published:** 2023-11-18

**Authors:** Kaisheng Yuan, Di Hu, Xiaocong Mo, Ruiqi Zeng, Bing Wu, Zunhao Zhang, Ruixiang Hu, Cunchuan Wang

**Affiliations:** 1 Department of Metabolic and Bariatric Surgery, the First Affiliated Hospital of Jinan University, Jinan University, Guangzhou, 510000 Guangdong China; 2 The Guangdong-Hong Kong-Macao Joint University Laboratory of Metabolic and Molecular Medicine, Jinan University, Guangzhou, 510000 Guangdong China; 3 Department of Neurology and Stroke Centre, the First Affiliated Hospital of Jinan University, Jinan University, Guangzhou, 510000 Guangdong China; 4 Department of Oncology, the First Affiliated Hospital of Jinan University, Jinan University, Guangzhou, 510000 Guangdong China; 5 Department of Urology, the Second People’s Hospital of Yibin City, Yibin, 644000 Sichuan China

**Keywords:** Colon cancer, Oxidative stress, Prognosis model, Bioinformatics analysis, SERPINE1

## Abstract

**Background:**

Colon cancer (CC) is a prevalent malignant tumor that affects the colon in the gastrointestinal tract. Its aggressive nature, strong invasiveness, and rapid progression make it a significant health concern. In addition, oxidative stress can lead to the production of reactive oxygen species (ROS) that surpass the body's antioxidant defense capacity, causing damage to proteins, lipids, and DNA, potentially promoting tumor development. However, the relationship between CC and oxidative stress requires further investigation.

**Methods:**

We collected gene expression data and clinical data from 473 CC patients from The Cancer Genome Atlas (TCGA) dataset. Additionally, we obtained 433 oxidative stress genes from Genecards (https://www.genecards.org/). Using univariate, multivariate, and LASSO Cox regression analyses, we developed predictive models for oxidative stress-related genes in CC patients. To validate the models, we utilized data from the Gene Expression Omnibus (GEO) database. We assessed the accuracy of the models through various techniques, including the creation of a nomogram, receiver operating characteristic curve (ROC) analysis, and principal component analysis (PCA). The Cytoscape program was utilized to identify hub genes among differentially expressed genes (DEGs) in tumor patients using the TCGA dataset. Subsequently, we conducted survival analysis, clinical relevance analysis, and immune cell relevance analysis for the intersected genes obtained by combining the hub genes with the genes from the predictive models. Moreover, we investigated the mRNA expression and potential functions of these intersected genes using a range of experimental approaches.

**Results:**

In both the TCGA and GSE17538 datasets, patients classified as high-risk had significantly shorter overall survival compared to those in the low-risk group (TCGA: p < 0.001; GSE17538: p = 0.010). As a result, we decided to further investigate the role of SERPINE1. Our survival analysis revealed that patients with high expression of SERPINE1 had a significantly lower probability of survival compared to those with low expression (p < 0.05). Additionally, our clinical correlation analysis showed a significant relationship between SERPINE1 expression and T, N, and M stages, as well as tumor grade. Furthermore, our immune infiltration correlation analysis demonstrated notable differences in multiple immune cells between the high- and low-expression groups of SERPINE1. To validate our findings, we conducted experimental tests and observed that knocking down SERPINE1 in colon cancer cells resulted in significant reductions in cell viability and proliferation. Interestingly, we also noticed an increase in oxidative stress parameters, such as ROS and MDA levels, while the levels of reduced GSH decreased upon SERPINE1 knockdown. These findings suggest that the antineoplastic effect of silencing SERPINE1 may be associated with the induction of oxidative stress.

**Conclusion:**

In conclusion, this study introduces a new approach for the early diagnosis and treatment of CC, and further exploration of SERPINE1 could potentially lead to a significant advancement.

**Supplementary Information:**

The online version contains supplementary material available at 10.1007/s12672-023-00833-w.

## Introduction

Colon cancer (CC) is a malignant tumor that originates from the mucosal epithelium of the colon. It accounts for over 1 million new cases worldwide each year, posing a serious threat to human health and survival [1]. CC has the second-highest mortality rate and the third-highest prevalence rate among all tumors [2]. While the incidence rate is decreasing among the elderly, it is increasing in younger individuals, causing the overall CC population to become younger [3]. Despite recent advancements in surgery, radiotherapy, targeted therapy, and immunotherapy, the 5-year survival rate for CC remains at 40–60% [4]. Early detection of CC is challenging as early symptoms are often not noticeable, and standard tumor markers have low sensitivity and specificity. Therefore, it is crucial to study diagnostic and prognostic biomarkers related to CC in order to improve the prognosis for individuals with this disease.

Oxidative stress is the physiological and pathological response of cells and tissues to the generation of reactive oxygen radicals (ROS) and reactive nitrogen radicals (RNS) in both internal and external environments [5]. An imbalance of oxidative stress can lead to the oxidation of nucleic acids, proteins, and lipids by ROS, promoting malignancy. Damaged DNA then enters the cytoplasm, triggering an interferon-mediated innate immune response that further stimulates ROS production. This creates a vicious cycle that maintains the inflammatory environment of the tumor [6, 7]. Tumor cells often have higher levels of ROS compared to normal tissues due to abnormal activation of oncogenes, inactivation of tumor suppressor genes, and metabolic reprogramming induced by hypoxia in the tumor microenvironment [8, 9]. Over 20 years ago, Haklar et al. observed a significant increase in ROS levels in CC tumor tissues through chemiluminescence analysis [10]. Other studies have investigated the association between genetic variants in antioxidant protective mechanism genes and the risk of CC [11]. Additionally, chronic inflammation caused by infections can increase ROS production in the colon, leading to oxidative stress in the colonic mucosa and impacting colon carcinogenesis [12]. Additionally, chronic inflammation caused by infections can increase ROS production in the colon, leading to oxidative stress in the colonic mucosa and impacting colon carcinogenesis [13]. However, further exploration is needed to understand the critical biological pathways regulated by oxidative stress and investigate the role of epigenetic factors in the pathogenesis of CC.

Therefore, the identification of prognostic genes and novel signature molecules associated with oxidative stress in colon cancer may provide valuable insights for the development of anti-CC strategies.

## Materials and methods

### Acquisition of mRNA expression data and clinical data

Clinical information, including general information, stage, and prognosis of CC samples, was obtained from The Cancer Genome Atlas (TCGA) (https://portal.gdc.cancer.gov/). As of October 2022, mRNA transcriptome data and connected medical records of 473 CC patients were obtained from the TCGA database. GSE17538 gene expression profiles were acquired from Gene Expression Omnibus (GEO) (https://www.ncbi.nlm.nih.gov/gds/), which included comprehensive transcriptome data for 238 patients with CC (platform: GPL570). Supplementary Table S1 provides detailed information on the patients.

### Identification of differentially expressed oxidative stress genes (DEOSGs)

A total of 433 oxidative stress protein domains with correlation scores ≥ 10 were collected from GeneCards (https://www.genecards.org) **(**Supplementary Table S2**)**. The FPKM values were used to standardize the expression data of the TCGA dataset for comparison, and gene expression related to oxidative stress was retrieved [14]. The extracted genes were then filtered using the 'limma' package based on a false discovery rate (FDR) < 0.05 and |log2 fold change (FC)|≥ 1.5, resulting in the identification of DEOSGs in CC tissues [15, 16]. The 'pheatmap' package was used to draw the heatmap and volcano plot of DEOSGs in CC [17].

### Univariate Cox regression analysis of prognostic-related oxidative stress genes in CC

The "limma" package was used to combine DEOSGs expression data and survival data, and univariate Cox regression analysis was performed using the "survival" and "survminer" packages. Hazard ratio (HR) and P-values of the DEOSGs were calculated, and genes with a P-value < 0.05 were considered significantly expressed genes. These genes were further classified based on HR > 1, with genes having HR > 1 considered high-risk genes and genes with HR < 1 considered low-risk genes. Somatic mutation data of CC were obtained from TCGA, and the 'maftools' package was employed to examine the mutation data and create oncoplot features [18]. The 'somatic Interactions' function was used to analyze the co-occurrence between the univariate significantly expressed genes and generate a co-occurrence graph [19].

### Construction of a prognostic model for oxidative stress-related genes

To identify key oxidative stress genes that independently affect the prognosis of CC, we conducted a comprehensive analysis. First, we included significantly expressed genes in a univariate analysis and then performed a multivariate Cox regression analysis. Next, we utilized the LASSO regression model (using the R package 'glmnet') to further narrow down the candidate genes from the TCGA database. Subsequently, we created a prognostic model and calculated risk scores by normalizing the TCGA expression data. The formula used to calculate the risk score for each sample was: risk score = Σ(Xi * Yi) (X: the regression coefficient, Y: oxidative stress-related genes expression value).

### Evaluation of the prognostic model for CC based on oxidative stress-related genes

Patients with CC in the TCGA database were categorized into low-risk and high-risk groups based on their median risk scores. Principal component analysis (PCA) was then conducted using the 'prcomp' function in the 'stats' R package. The overall survival of patients in both groups was compared using Kaplan–Meier analysis. Additionally, the prognostic risk model's validity was assessed using the GSE17538 dataset. The samples in this dataset were divided into high-risk and low-risk groups based on their risk scores, and the overall survival between the two patient groups was compared.

Clinical progression-free survival (PFS) analysis was performed by combining pan-cancer clinical data with data on DEGs from the TCGA database, using the 'survival' and 'survminer' packages [20]. Clinical receiver operating characteristic (ROC) analysis, as well as 1-, 3-, and 5-year ROC analysis, were conducted using the 'Survival', 'survminer,' and 'timeROC' packages. The area under the curve (AUC) was calculated. Furthermore, age, gender, cancer grade, and TNM stage differential analyses in the TCGA database were performed using the 'ggpubr' software.

Relevant clinical information was extracted for patients in the TCGA cohort. These variables, along with risk scores, were investigated using a regression model. Univariate and multivariate Cox regression models were employed to analyze the relationship between risk scores, clinical characteristics, and overall survival.

Based on the age, sex, tumor grade, TNM stage, and risk scores of patients in the TCGA database, a nomogram and its corresponding calibration diagram were constructed using the 'rms' package in R software. The ROC curve analysis for the nomogram was performed using the 'survival', 'survminer', and 'timeROC' packages. In the univariate and multivariate Cox regression analyses, patients' age, sex, histological grade, and nomogram risk scores were included. P-values and HR values were calculated for each factor, respectively.

### Analysis of immune cell infiltration

In the TCGA cohort, individuals were categorized into low- and high-risk groups according to their median risk ratings. The infiltration of various immune cell types in TCGA samples from these groups was predicted using the CIBERSORT method and MCP-counter algorithm [21]. Immune cells with a p-value of less than 0.05 were chosen, and the expression of 22 immune cell types in the high- and low-risk groups was analyzed using the "reshape2" and "ggpubr" packages.

### Gene set variation analysis

The gene set 'c2.cp.kegg.v7.0.symbols.gmt' from the Molecular Signatures Database (MSigDB) was used as a reference gene set. The individuals were divided into low- and high-risk groups based on the median expression of DEOSGs in TCGA. Gene Set Variation Analysis (GSVA) was performed using the 'GSVA' and 'GSEABase' packages to identify mechanisms significantly correlated with the prognostic expression of oxidative stress-related genes (P < 0.05).

### DEGs in TCGA and functional enrichment analysis

To further analyze the DEGs, we divided individuals with CC in the TCGA cohort into two subgroups based on their median risk scores. The DEGs were then compared between the low-risk and high-risk groups using specific criteria (|Log2FC|≥ 1 and FDR < 0.05). We performed functional enrichment analysis of the DEGs using the 'clusterProfiler' software, which included independent gene ontology (GO) and Kyoto Encyclopedia of Genes and Genomes (KEGG) analyses.

### Construction of Protein–Protein Interaction (PPI) networks for DEGs and identification of hub genes

The DEGs were analyzed using the STRING software (https://www.string-db.org) to construct protein–protein interaction (PPI) networks. The networks were visualized and further analyzed using the Cytoscape software. Hub genes were identified using the cyto-Hubba plug-in (http://apps.cytoscape.org/apps/cytohubba) [22]. The top 15 genes with the highest node degree were selected as hub genes.

### Survival analysis, clinical correlation analysis, and immune cell correlation analysis of hub genes

Subjects from the TCGA cohort with CC were divided into two groups based on the expression levels of the hub gene: low-expression and high-expression groups. Only CC samples with available medical information were included for analysis. The survival differences between the two groups were compared using the 'survival' and 'survminer' packages. Clinical features associated with the expression of the hub gene were visualized using the 'ggpubr' package. The expression levels of 22 immune cells in the low-expression and high-expression groups were examined using the 'reshape2' and 'ggpubr' packages after conducting significance screening (P < 0.05).

### Cell culture and CC specimens

The CC cell lines (SW480, CACO-2, and HT29) and human intestinal epithelial cells (NCM460) were obtained from Dr. Yunlong Chen. The CC cells were cultured in DMEN medium (Gibco). From August 2021 to October 2022, a total of 28 pairs of CC tissues (T) and normal para-tumor tissues (N) were collected during surgeries at the First Affiliated Hospital of Jinan University. The inclusion of para-tumor tissue allowed for the comparison between cancerous and adjacent non-cancerous tissues to assess differences in SERPINE1 expression. All tissue samples were immediately frozen in liquid nitrogen and stored at −80 °C until extraction. The tissue specimens were confirmed through postoperative histopathological examination. The study was approved by the Ethics Committee of the First Affiliated Hospital of Jinan University, and informed consent was obtained from all participants.

### Cell transfection

Si-SERPINE1 and its negative control (si-ctrl) were obtained from GenePharma (Shanghai, China). SW480 and CACO-2 cells were cultured in multiple 24-well plates. Lipo3000 (Invitrogen, USA) was used to transfect the two CC cells with plasmid when they reached approximately 80% confluence.

### CCK-8 assay

CC cells (SW480 and CACO-2) were seeded in 96-well plates and incubated at a specialized cell incubator for four time points (0-72 h). CCK-8 (Life-iLab; China) was then added to the appropriate groups in suitable proportions. The CC cells were subsequently incubated for 1–2 h at 37 °C and 5% CO_2_. The absorbance at 450 nm was measured using an enzyme labeling instrument.

### Colony formation assay

SW480 and CACO-2 cells were seeded in 6-well plates and incubated for 24 h. After transfection, the cells were cultured for 2 weeks at 37 °C and 5% CO2, with the medium being changed every 3 days. Upon completion, the colonies were fixed with 4% paraformaldehyde (1 mL/well) for 15 min and then stained with 0.1% crystal violet (1 mL/well) for 20 min. Finally, the software 'ImageJ' was utilized to count all the colonies.

### Western blot

The SW480 and CACO-2 cells that had been treated were collected and lysed in RIPA buffer. The proteins of interest (20–30 µg/lane) were then separated using SDS-PAGE (12%, 80 min) and transferred onto appropriately sized PVDF membranes. The membranes were blocked with experimental quick sealing fluid (Life-iLab; AP36L118; China) for 30 min and then incubated overnight at 4 °C with anti-SERPINE1 (ab270058; 1:1000; USA) and β-actin (#4970; 1:4000; CST; USA) antibodies. The following day, the membranes were incubated with the appropriate secondary antibody and visualized using a double-enhanced ECL kit (Data Invention Blotech; DIB052; China).

### RT − qPCR analysis

Total RNA was extracted from SW480 and CACO-2 cells using Trizol (Beyotime, China). The RNA was then reverse-transcribed into complementary DNA (cDNA) using the SuperScript VILO cDNA Kit (Thermo Fisher Scientific, Inc.). The qRT-PCR results were analyzed using the 2-ΔΔCt method. The primer sequences used for SERPINE1 were as follows: Forward primer: 5′-GGGTTTTCGTGGTTCACATCC-3ʹ; Reverse primer: 5′-CTAGACGCTGGCTCCTCAGTA-3ʹ. For GAPDH, the primer sequences used were: Forward primer: 5′-ATCACTGCCACCCAGAAGAC-3ʹ; Reverse primer: 5′-ACACATTGGGGGTAGGAACA-3ʹ.

### Immunohistochemistry (IHC)

The human tissues were fixed in 4% paraformaldehyde for 15 min, embedded in paraffin, and cut into 4 μm sections. After dewaxing and dehydration, the antigens were retrieved. Subsequently, the sections were treated with 3% hydrogen peroxide for 20 min and blocked with 5% BSA at room temperature for 15 min. Following this, the sections were incubated overnight at 4 °C with anti-SERPINE1 (1:100) antibody. The sections were then stained with a color-developing agent for 3–15 min, washed, restained, dehydrated, transparentized, and sequentially sealed. Finally, the sections were observed and photographed under a light microscope.

### Determination of ROS production

SW480 and CACO-2 cells were seeded in 6-well plates and incubated for 24 h. Following transfection, the cells were incubated at 37 °C for approximately 30 min with serum-free media containing DCFH-DA (Beyotime, China). Flow cytometry was used to analyze the plates and obtain the outcomes of the treated cells, which were then preserved.

### MDA and GSH assay

MDA and GSH levels were measured using the MDA Assay Kit (Beyotime; S0131; China) and GSH Assay Kit (Nanjing Jiancheng Bioengineering Institute; A006-2-1; China) according to the provided directions.

### Statistical analysis

Gene expression in CC and para-tumor tissues was compared using univariate ANOVA, while categorical variables were compared using Pearson chi-square tests. Overall survival between the two patient groups was assessed using stratified log-rank testing and Kaplan–Meier analysis. The independent prognostic efficacy of the risk model was evaluated using univariate and multivariate Cox regression models. R program (v4.2.1) and the Perl language (version 5.30.0) were used for all statistical analyses. Statistical significance was defined as P < 0.05.

## Results

### Identification of DEOSGs

The study design is illustrated in Supplementary Figure S1. Using the TCGA database, we compared the expression of 433 genes related to oxidative stress in 473 CC tissues. We identified 36 down-regulated genes and 29 up-regulated genes, which had a false discovery rate (FDR) < 0.05 and a |log2 fold change (FC)|≥ 1.5. Comprehensive data on the differentially expressed oxidative stress-related genes (DEOSGs) can be found in Supplementary Table S3. The top 50 genes with the greatest increase and decrease are shown in Supplementary Figure S2A. Additionally, all DEOSGs are depicted in the volcano plot (Supplementary Figure S2B).

### Screening of prognostic oxidative stress-related genes in CC

Through a univariate Cox regression analysis in the TCGA database, we have identified 23 prognostic oxidative stress-related genes for CC (P < 0.05). Genes with a hazard ratio (HR) less than 1 are considered low-risk genes, while genes with HR greater than 1 are considered high-risk genes. The 95% confidence intervals for the HR values are shown in brackets in supplementary Figure S3A. The waterfall plot illustrates variations in the distribution of somatic mutations in the 23 DEOSGs (supplementary Figure S3B), and the co-occur plot indicates the co-occurrence between these 23 DEOSGs (supplementary Figure S3C).

### Construction and evaluation of a prognostic model for CC based on oxidative stress-related genes

Through LASSO regression analysis, we identified 16 genes (NGF, IL13, RPS6KA5, TERT, CDKN2A, SERPINE1, CD36, PPARGC1A, ACADL, ACOX1, POMC, BDNF, MSRA, DDIT3, GSTM1, CPT2) based on the best λ value to construct a prognostic model (Supplementary Figure S4A–B). The risk score is calculated using the following formula: risk score = (0.0708 * NGF exp.) + (−2.4907 * IL13 exp.) + (−0.1846 * RPS6KA5 exp.) + (0.7210 * TERT exp.) + (0.1351 * CDKN2A exp.) + (0.1102 * SERPINE1 exp.) + (0.1850 * CD36 exp.) + (−0.3145 * PPARGC1A exp.) + (0.3644 * ACADL exp.) + (−0.0843 * ACOX1 exp.) + (0.1684 * POMC exp.) + (0.5691 * BDNF exp.) + (−0.2211 * MSRA exp.) + (0.2156 * DDIT3 exp.) + (−0.1499 * GSTM1 exp.) + (−0.2575 * CPT2 exp.). We divided the 473 individuals with colorectal cancer into low- and high-risk groups by using the median score obtained from the risk score method. Subsequently, the patients with different risks were further divided into two groups based on PCA for oxidative stress genes and model genes (Supplementary Figure S4C–D). Analysis of the TCGA database revealed a significant difference in overall survival between the low- and high-risk groups (P < 0.001) (Supplementary Figure S4E). To validate the model, we used the GSE17538 database as an external validation group. The analysis showed a statistically significant difference in overall survival between the low- and high-risk groups (P = 0.010) (Supplementary Figure S4F). Furthermore, in the TCGA cohort, the high-risk group exhibited significantly poorer PFS compared to the low-risk group (P < 0.001) (Supplementary Figure S4G).

To evaluate the risk signature as an independent predictive variable, we used univariate and multivariate Cox regression models. The results showed that the risk score was an independent predictor of a worse patient prognosis (HR = 3.461, 95% CI 2.552–4.693) based on univariate Cox regression analysis (Supplementary Figure S5A). After adjusting for other confounding variables, the multivariate Cox regression analysis demonstrated that the risk score remained a significant predictive variable for individuals with CC (HR = 2.973, 95% CI 2.124–4.162) (Supplementary Figure S5B). We assessed the sensitivity and specificity of the model using ROC analysis, and found that the areas under the ROC curves for 1, 3, and 5 years were 0.706, 0.730, and 0.781, respectively (Supplementary Figure S5C). Over five years, the prognostic model showed superior predictive accuracy compared to other clinical characteristics in the TCGA cohort (Supplementary Figure S5D).

To determine the association between each clinicopathological characteristic and the risk score, we analyzed age, sex, tumor grade, and TNM stage in the TCGA cohort. The results indicated that the risk score was positively associated with T-stage, except for T1 and T2 stages (Supplementary Figure S5E). Patients with a higher N stage (Supplementary Figure S5F) or M1 stage (Supplementary Figure S5G) of CC were significantly associated with higher risk scores. Regarding tumor grade, the risk score was positively associated with tumor grade, except for stages III and IV (Supplementary Figure S5H). However, there was no significant relationship between the risk score and age or sex in individuals with CC (all P > 0.05).

### Construction and validation of a prognostic nomogram for CC based on oxidative stress-related genes

Based on the findings of the multivariate Cox regression analysis in the TCGA cohort, we constructed a nomogram using independent risk variables including age, sex, tumor grade, TNM stage, and risk scores (Supplementary Figure S6A). To evaluate the nomogram's prediction ability, we plotted clinical ROC curves and calibration curves. The calibration curve (Supplementary Figure S6B) showed a high consistency between the risk predicted by the nomogram and the detected 1-, 3-, and 5-year survival rates. The nomogram also exhibited superior predictive accuracy compared to other clinical variables over a 5-year period, as indicated by the clinical ROC curve (AUC = 0.819) (Supplementary Figure S6C). Moreover, the Nomo risk score showed a significant association with overall survival according to the Univariate Cox regression analysis [HR = 1.309 (1.238–1.383), P < 0.001, Supplementary Figure S6D]. Additionally, the Nomo risk score was identified as an independent risk factor for overall survival in subjects with CC using multivariate Cox regression analysis [HR = 1.167 (1.075–1.266), P < 0.001, Supplementary Figure S6E].

### Differential analysis of immune cells and GSVA analysis

The analysis of immune cell differences revealed significant variations in the expression of B memory cells, plasma cells, T CD4 memory resting cells, NK cells, Macrophages M0, Dendritic cells resting cells, Dendritic cells activated cells, and Eosinophils between the high- and low-risk groups of the TCGA cohort (P < 0.05) (Supplementary Figure S7A). The MCP-counter algorithm also identified significant differences in the expression of NK cells, myeloid dendritic cells, myeloid dendritic cells, and fibroblasts between the low-risk and high-risk groups (Supplementary Figure S7B). Furthermore, the GSVA analysis revealed that the high-risk group of the TCGA cohort had significantly elevated expression levels of the circadian rhythm mammal, basal cell carcinoma, glycosaminoglycan biosynthesis, chondroitin sulfate, and ECM receptor interaction mechanisms (Supplementary Figure S7C).

### Screening and functional enrichment analysis of DEGs in TCGA cohort of CC

Based on the median risk score mentioned above, a differential analysis was conducted on individuals with CC in the TCGA cohort, resulting in the identification of 70 genes. Among these genes, 17 were found to be down-regulated while 53 were up-regulated (Supplementary Table S4). The GO analysis of these 70 DEGs in CC indicated that biological processes (BP) were mainly associated with muscle contraction and intermediate filament-based mechanisms. CC were dominated by collagen-containing extracellular matrix and endoplasmic reticulum lumen. In terms of molecular function (MF), receptor ligand and signaling receptor activator activity were found to be predominant (Supplementary Figure S8A-B). Furthermore, the KEGG pathway analysis revealed that the main signaling pathways involved were ECM-receptor interaction, Human papillomavirus infection, and PI3K-Akt signaling pathway (Supplementary Figure S8C-D).

### Identification of hub genes and prognostic analysis of CDKN2A and SERPINE1

**Fig. 1:**
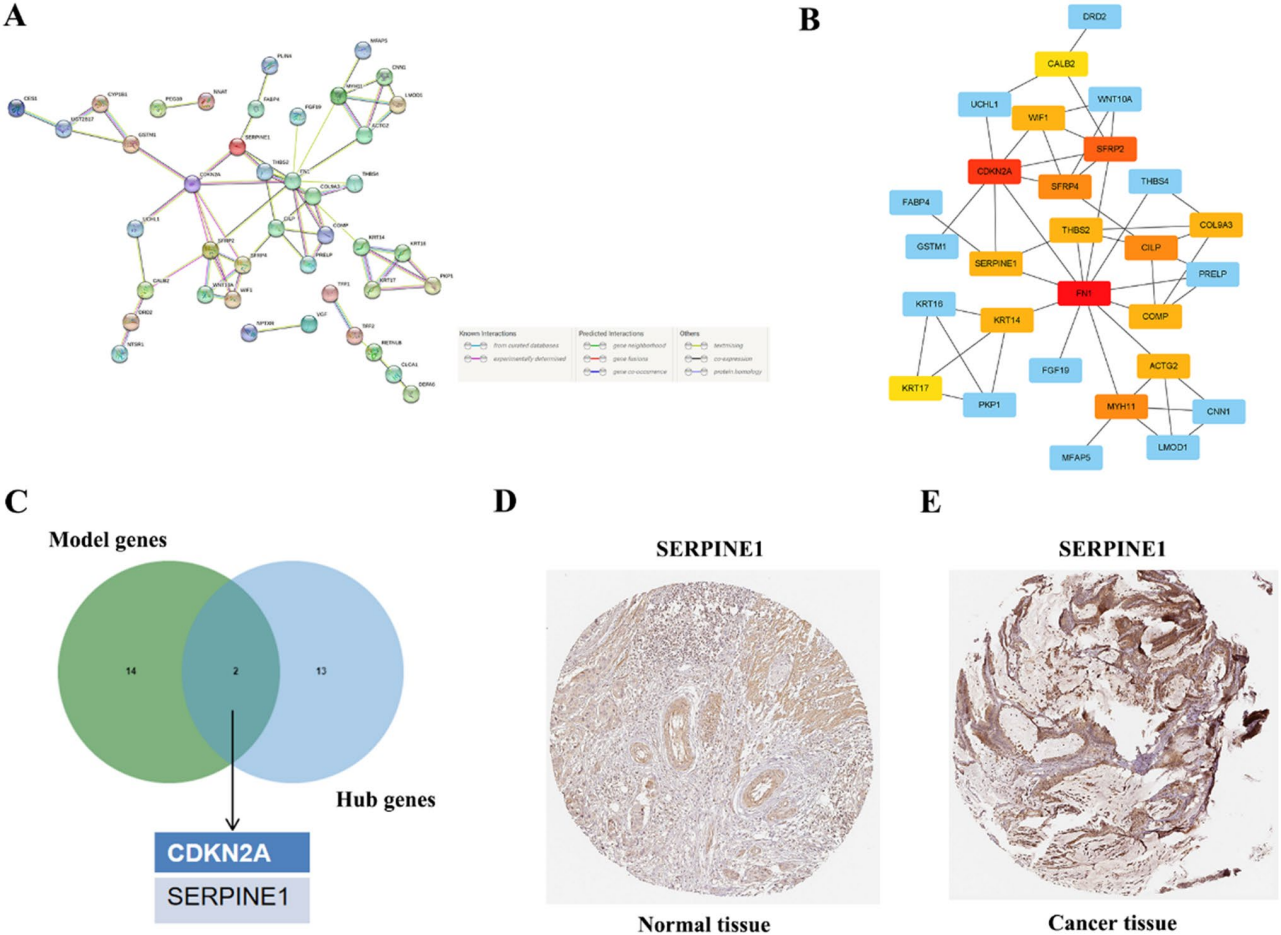
Construction of PPI networks and identification of hub genes. **A** The PPI network showed the interactions of the DEGs (interaction score = 0.4). **B** Visualization of the PPI network and the candidate hub gene according to the EPC ranking. **C** A Venn diagram shows the number of overlapped genes between the top ten hub genes and the genes involved in the construction of the prognostic model for CC. **D**–**E** Immunohistochemical staining of SERPINE1 gene in normal tissue (**D**) and cancer tissue (**E**) of CC in HPA database

Using the STRING software, it was discovered that out of the 70 DEGs identified in CC, 63 were involved in constructing PPI networks. The resulting PPI network consisted of 62 edges, with an average node degree of 1.97 and an average local clustering coefficient of 0.393. Statistically, the difference in this PPI network was found to be significant (P < 0.05) (Fig. 1A). To identify the top 15 hub genes based on node degree, the Cyto-Hubba plug-in was utilized, resulting in the selection of FN1, CDKN2A, SFRP2, MYH11, SFRP4, CILP, COL9A3, SERPINE1, WIF1, COMP, ACTG2, KRT14, THBS2, CALB2, and KRT17 (Fig. 1B). Furthermore, by intersecting the top 15 hub genes with the genes used to construct the predictive model for CC, CDKN2A and SERPINE1 were also identified (Fig. 1C). The Human Protein Atlas (HPA, https://www.proteinatlas.org/) offers an IHC-based assay for relative protein abundance [23]. In terms of oxidative stress, SERPINE1 exhibited a higher coefficient than CDKN2A (12.33 vs. 11.43). To examine the protein expression of SERPINE1 in CC and normal tissues, HPA data was utilized, which revealed a noticeable accumulation of SERPINE1 in CC tissues (Fig. 1D, E).

### Clinical correlation analysis and immune infiltration analysis of SERPINE1

**Fig. 2:**
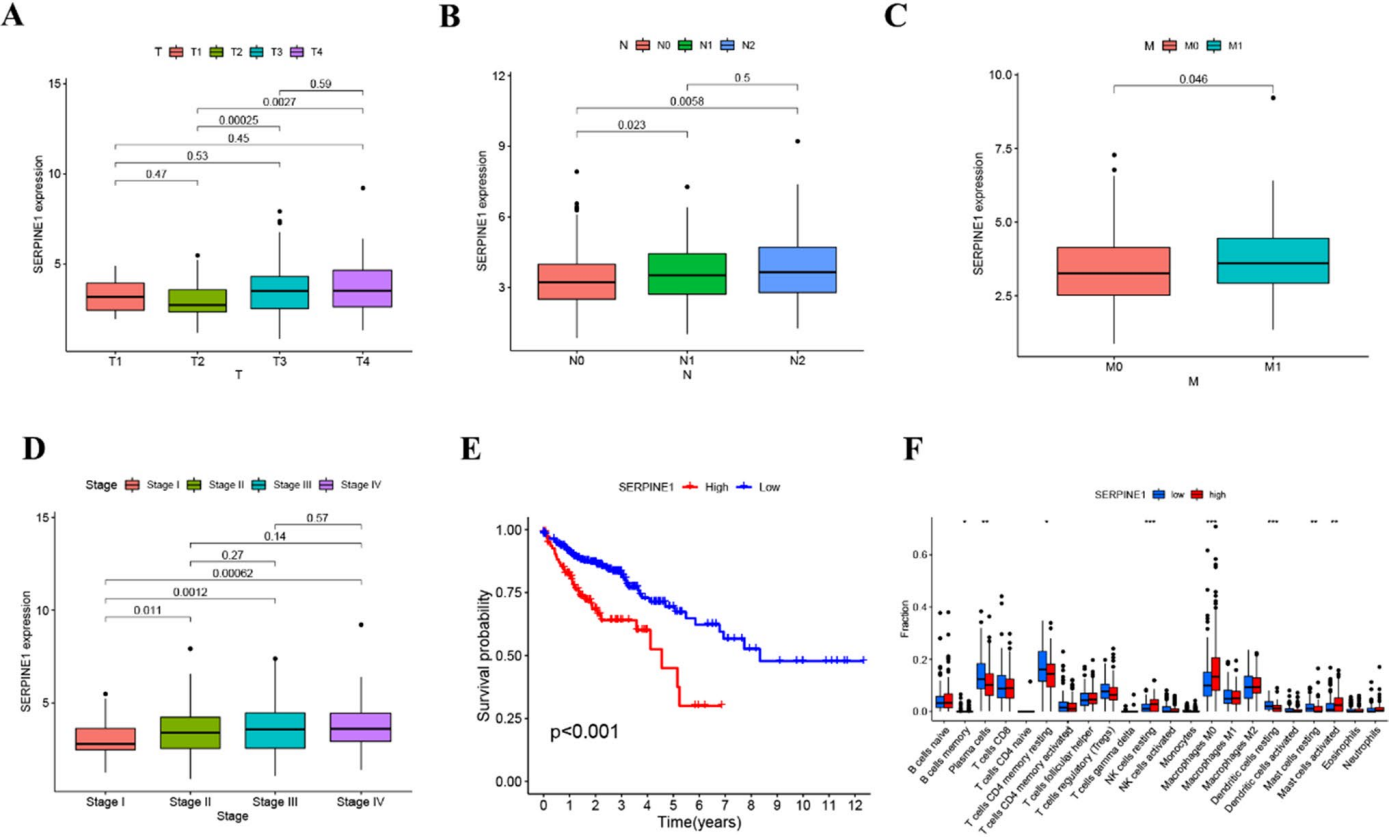
Clinical correlation analysis and immune infiltration analysis of SERPINE1. **A** The relationship between SERPINE1 expression and T stage in TCGA cohort. **B** The relationship between SERPINE1 expression and N stage in TCGA cohort. **C** The relationship between SERPINE1 expression and M stage in TCGA cohort. **D** The relationship between SERPINE1 expression and tumor stage in TCGA cohort. **E** Kaplan–Meier curves for comparison of the overall survival between SERPINE1 low-and high-expression groups in the TCGA database (*P* < 0.001). **F** CIBERSORT score of 22 immune cell infiltrations among TCGA samples of SERPINE1 low-expression and high-expression groups

The relationship between SERPINE1 expression and the clinicopathological characteristics of subjects with CC in the TCGA dataset was investigated. The results revealed a significant correlation between SERPINE1 expression and the cancer grade and TNM stages of individuals with CC. SERPINE1 expression was lower in T2 patients compared to T3 or T4 patients (P = 0.00025 and P = 0.0027, respectively) (Fig. 2A). Additionally, SERPINE1 expression was lower in N0 patients compared to N1 or N2 patients (P = 0.023 and P = 0.0058, respectively) (Fig. 2B). Moreover, SERPINE1 expression was lower in M0 patients compared to M1 patients (P = 0.046) (Fig. 2C). In terms of tumor stage, SERPINE1 expression was lower in Stage I patients compared to Stages II, III, or IV patients (P = 0.011, 0.0012, and 0.00062, respectively) (Fig. 2D). However, there was no correlation between SERPINE1 expression and gender or age (P = 0.60 and 0.32, respectively). Survival analysis using the Kaplan–Meier method revealed that patients with reduced SERPINE1 expression had significantly longer overall survival compared to those with elevated expression (Fig. 2E). To examine the connection between SERPINE1 and immune cell infiltration, a correlation study was conducted to assess the relationship between SERPINE1 expression and the abundance of 22 immune cell types. The results showed that B cells naive, macrophages M0, and activated mast cells were significantly more abundant in the high-expression group of SERPINE1, while plasma cells, T cells CD4 memory resting, NK cells resting, dendritic cells resting, and mast cells resting were more abundant in the low-expression group (Fig. 2F).

### The expression of SERPINE1 in CC tissues and cell lines

**Fig. 3:**
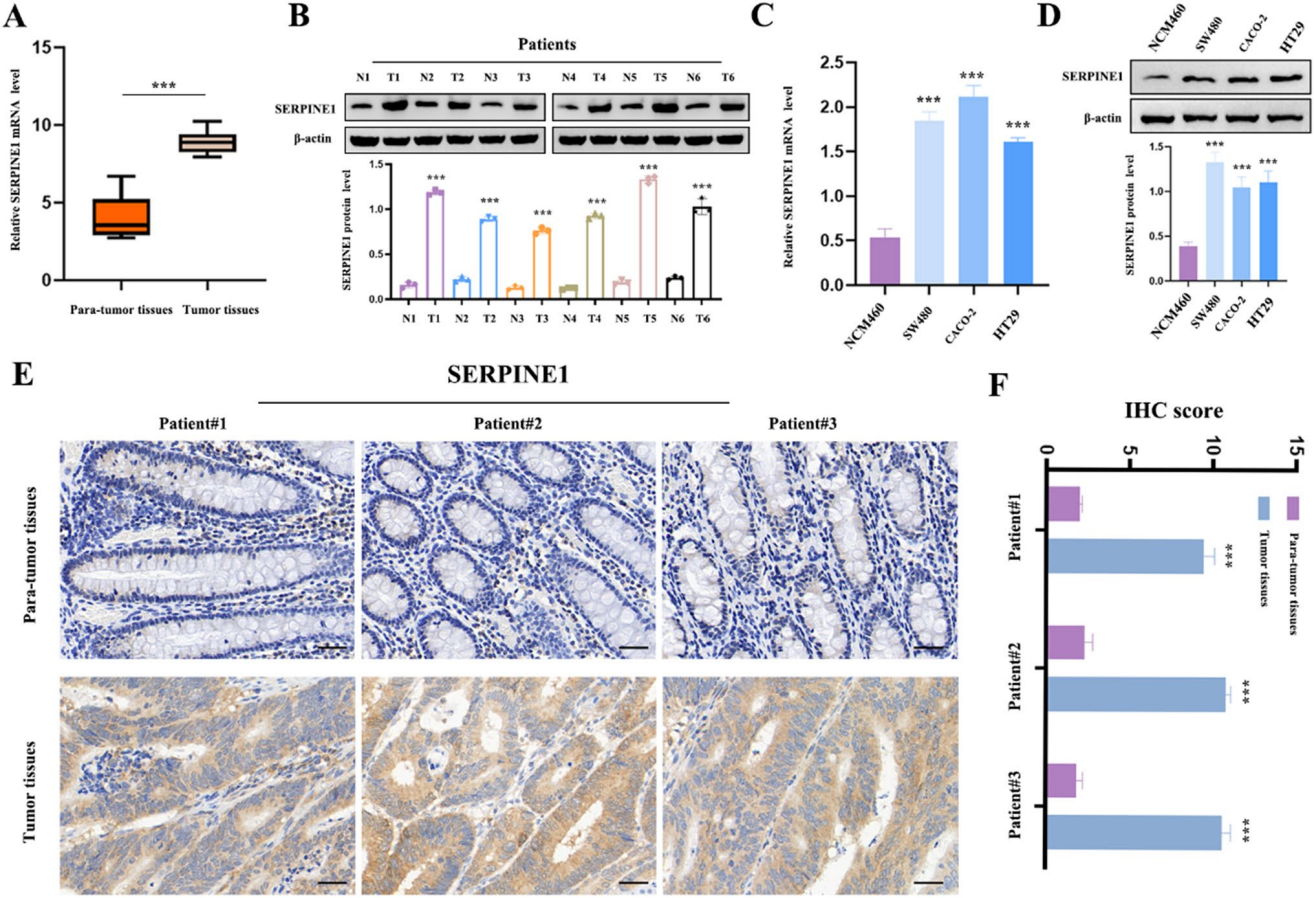
The expression of SERPINE1 in CC tissues and cell lines. **A** SERPINE1 mRNA expression in CC specimens relative to para-tumor tissues as detected by RT-qPCR. **B** SERPINE1 protein level in 6 CC patients relative to para-tumor tissues as conducted by western blot. **C** SERPINE1 expression in 3 different CC cell lines (SW480, CACO-2, and HT29) relative to NCM460 was conducted by RT-qPCR. **D** SERPINE1 expression in 3 different CC cell lines (SW480, CACO-2, and HT29) relative to NCM460 was conducted by western blot. **E**–**F** Immunohistochemical analysis of SERPINE1 expression in CC patients. *p < 0.05; **p < 0.01; ***p < 0.001

Following the comprehensive online machine learning analysis, RT-qPCR and western blot were conducted to confirm the significantly higher levels of SERPINE1 mRNA and protein in CC tissues compared to para-tumor tissues (Fig. 3A, B). In order to validate these findings, three different CC cell lines (SW480, CACO-2, and HT29) were selected for in vitro experiments, with human intestinal epithelial cells (NCM460) serving as the control group. The results consistently demonstrated overexpression of SERPINE1 in the CC cancer cell lines (Fig. 3C, D). Furthermore, IHC analysis of human tissues provided additional confirmation of the prominent accumulation of SERPINE1 in tumor regions (Fig. 3E, F; Supplementary Figure S9).

### Effects of SERPINE1 knockdown on CC cell viability, proliferation, and oxidative stress

**Fig. 4:**
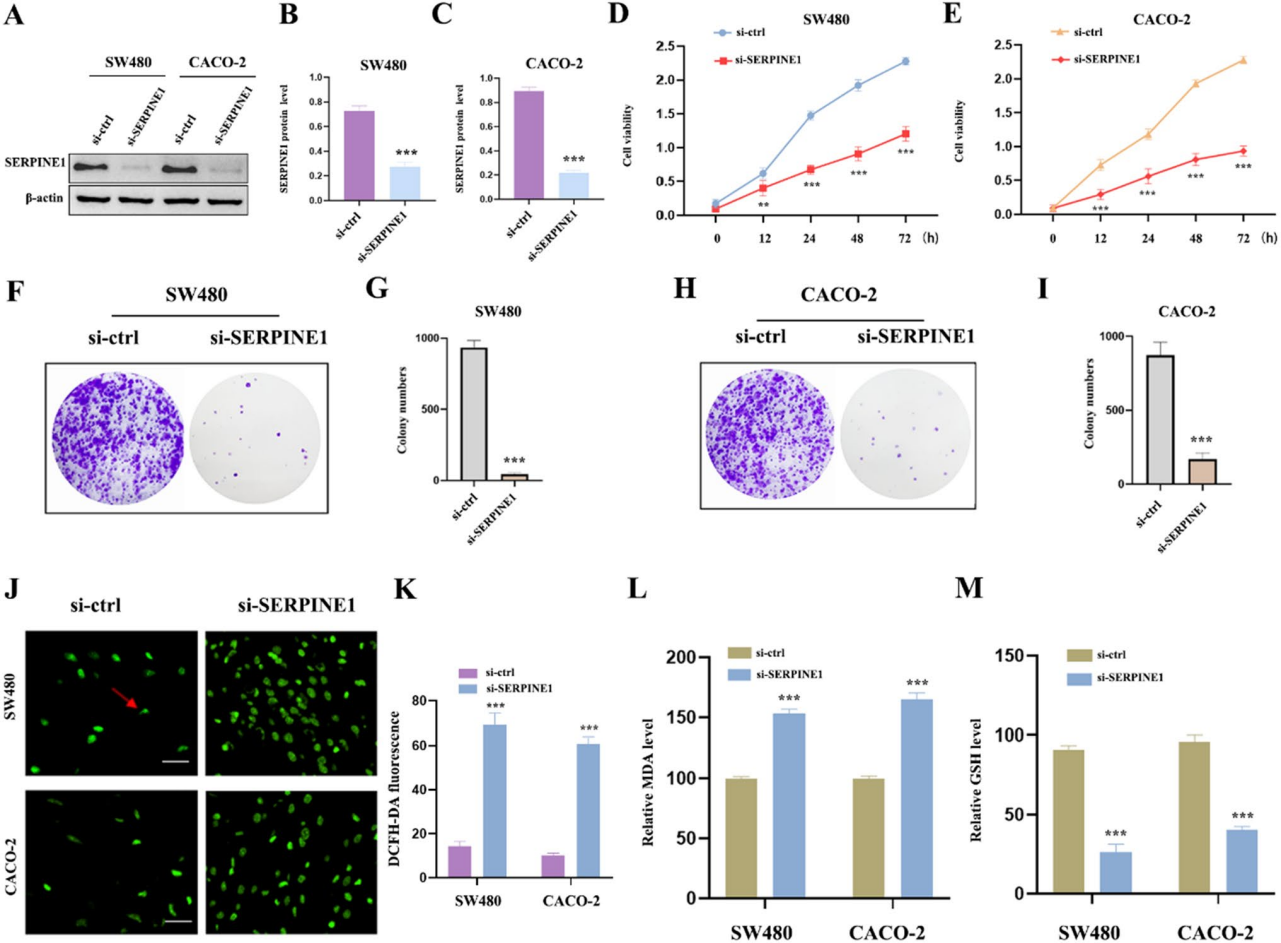
The effect of SERPINE1 knockdown on CC cell viability, proliferation as well as oxidative stress in vitro. **A** The efficiency of SERPINE1 knockdown (si- SERPINE1) was assessed by western blot in SW480 and CACO-2 cells. **B** Quantitative analysis of western blot in SW480 cell. **C** Quantitative analysis of western blot in CACO-2 cell. **D** The viability of SW480 cell was assessed by CCK-8 assays. **E** The viability of CACO-2 cell was assessed by CCK-8 assays. **F**–**G** The clone capacity of SW480 cell was evidenced by colony formation assay. **H**–**I** The clone capacity of CACO-2 cell was evidenced by colony formation assay. **J**–**K** ROS level of SW480 and CACO-2 cells. **L** MDA level of SW480 and CACO-2 cells. **M** GSH level of SW480 and CACO-2 cells. *p < 0.05; **p < 0.01; ***p < 0.001

Functional interference techniques were employed to examine the impact of SERPINE1 deletion on the behavior of CC cells, aiming to determine the specific role of SERPINE1 in the initiation and development of CC. Figure 4A–C demonstrate the successful transfection in CC cell lines. The results of CCK-8 analysis revealed that suppressing SERPINE1 significantly reduced cell viability in SW480 and CACO-2 cell lines (Fig. 4D, E). Similarly, the data from colony formation analyses indicated that the clone capacity of SW480 and CACO-2 cell lines was diminished after silencing SERPINE1 (Fig. 4F–I). These findings suggest that SERPINE1 plays a crucial role in promoting the progression of CC. Furthermore, it is well-established that oxidative stress is involved in the occurrence and development of cancers. Therefore, this study investigated the levels of ROS, MDA, and GSH in SW480 and CACO-2 cells (Fig. 4J–M), which revealed a significant increase in MDA and ROS levels and a noticeable decrease in GSH levels after transfecting SERPINE1 compared to the si-ctrl group. In summary, the anti-tumor effect of SERPINE1 knockdown on CC may be partly attributed to inducing oxidative stress.

## Discussion

CC is the most prevalent malignant tumor of the gastrointestinal system, ranking third among all malignant tumors in terms of incidence. The prognosis of CC is poor, and its pathogenesis remains poorly understood. As research on oxidative stress in intestinal diseases advances, there is increasing evidence from experimental and clinical data suggesting that ROS-mediated oxidative stress plays a vital role in CC development [11, 24]. Therefore, it is crucial to explore the function of oxidative stress-related genes in CC. This exploration may help elucidate the pathogenesis of CC, identify specific biomarkers, and discover therapeutic targets for patients with this condition.

The aim of this investigation was to identify prognostic markers for CC based on oxidative stress-related genes and develop a predictive model. Expression data from individuals with CC were obtained from the TCGA database, and a total of 204 differentially expressed oxidative stress genes (DEOSGs) were identified through differential expression analysis. Uni-variate Cox regression analysis using the survival package in the R program was performed to investigate the relationship between the expression of these genes and CC prognosis. This analysis resulted in the identification of 23 oxidative stress genes associated with CC prognosis. Subsequently, multivariate Cox regression analysis was conducted on these 23 genes, leading to the identification of 16 genes significantly associated with CC prognosis. These genes include NGF, IL13, RPS6KA5, TERT, CDKN2A, SERPINE1, CD36, PPARGC1A, ACADL, ACOX1, POMC, BDNF, MSRA, DDIT3, GSTM1, and CPT2. A predictive model for CC was developed using these 16 genes.

Each patient in the TCGA cohort was assigned a risk score using the Lasso regression model. The participants were divided into high- and low-risk groups based on the median value of the scores to assess the predictive value and reliability of the model. The survival study revealed that the prognosis of the high-risk group was significantly worse than that of the low-risk group. To validate the model, we also categorized patients with CC in GSE17538 into high- and low-risk groups based on their risk scores, which yielded the same result. The ROC analysis results showed that the AUC for 1-, 3-, and 5-year predictions were 0.706, 0.730, and 0.781, respectively, indicating excellent prognostic predictive ability of the model for CC. Univariate and multivariate Cox regression analyses identified the risk score as an independent predictive factor. The nomogram also demonstrated similar patterns, with survival, ROC, and univariate and multivariate regression analyses showing superior efficacy of the nomogram compared to other clinicopathological features.

Based on the median risk scores, differential analysis of CC subjects in the TCGA cohort was conducted to identify 70 differentially co-expressed genes with similar expression trends. Further analysis of the top 15 genes with the highest node degrees was performed using PPI networks. These 16 genes overlapped with the 16 genes involved in the model construction, leading to the identification of CDKN2A and SERPINE1. The HPA database revealed higher SERPINE1 protein expression in CC tissues compared to the healthy group [23]. Additionally, we observed that SERPINE1 had a higher oxidative stress coefficient than CDKN2A (12.33 vs. 11.43). Therefore, SERPINE1 was ultimately identified as the hub gene.

SERPINE1, a member of the serine protease inhibitor (serpin) superfamily, is primarily found in the gallbladder, liver, bladder, and placental tissues. It acts as a key suppressor of fibrinogen activator and plays a crucial role in regulating fibrinolysis [25, 26]. Moreover, SERPINE1 is significantly expressed in various tumor tissues and has been linked to cancer progression and metastasis [27]. Kim et al. conducted a study where they directly treated colon cancer cells with purine-based antiplatelet agents, confirming the increased expression of SERPINE1 in colon cancer. They observed that the overexpression of SERPINE1 enhanced cellular mobility in cancer cells, indicating its association with colon cancer metastasis [28]. Additionally, Chen et al. suggested that SERPINE1 may regulate the expression of VEGF and IL-6 through the JAK-STAT3 inflammatory and VEGF signaling pathways, thereby influencing GC cell invasion and migration [29]. Mazzoccoli et al. investigated SERPINE1 expression in 50 CC specimens, para-tumor tissues, and CC cell lines. They found that SERPINE1 expression was elevated in CC and highly proliferative CC cell lines, and it was also associated with tumor aggressiveness and invasiveness [30].

It was discovered that SERPINE1 hinders the tumor healing effects of miR148a-3p in CC, which includes cell growth and invasion [31]. Through univariate Cox regression and clinical correlation analyses, it was determined that high expression of SERPINE1 indicates a poorer prognosis, higher tumor staging, and grading. Wang et al. utilized the CIBERSORT method to evaluate the relationship between differential expression of SERPINE1 and immune cell infiltration. They ultimately identified SERPINE1 as a potential regulator of immune cell infiltration, capable of interacting with eight immune cell types, thereby reshaping the tumor microenvironment in colon cancer development and progression [32]. Our analysis of the relationship between SERPINE1 and 22 types of immune cells revealed that SERPINE1 is associated with numerous immune cells, suggesting its involvement in the immune infiltration process during CC development. These findings collectively suggest that SERPINE1 may play a role in CC progression and impact prognosis through its involvement in the regulation of oxidative stress. This study demonstrated that both SERPINE1 mRNA and protein levels were found to be overexpressed in human CC tumor tissues and cells. Functional studies were conducted using si-SERPINE1 transfection, which showed that interference with SERPINE1 significantly reduced the viability and growth of CC cells. Additionally, SERPINE1 inhibition led to a restoration of ROS and MDA accumulation and hindered the GSH content.

However, it is important to note that identifying the hub gene is only the initial step in screening biomarkers and therapeutic targets for CC. Further experiments are needed to validate the significance of SERPINE1 in CC development and prognosis, as well as to explore its molecular mechanism in regulating oxidative stress. Moreover, our findings underscore the intricate interplay between oxidative stress, SERPINE-1, and immune infiltration in CC. Further research is needed to elucidate the precise mechanisms underlying these relationships and their implications for CC progression and treatment.

## Conclusion

In this study, we conducted bioinformatics analysis and validation to screen 16 prognostic genes associated with oxidative stress in CC. These genes are NGF, IL13, RPS6KA5, TERT, CDKN2A, SERPINE1, CD36, PPARGC1A, ACADL, ACOX1, POMC, BDNF, MSRA, DDIT3, GSTM1, and CPT2. Using these 16 genes, we developed a prognostic model for CC that showed strong predictive value and reliability in determining patient outcomes. Additionally, our findings suggest that SERPINE1 may contribute to the development of CC through its regulation of oxidative stress. Therefore, SERPINE1 holds promise as a potential therapeutic target and a novel prognostic biomarker for CC.

### Supplementary Information

Additional file1 (TIF 9283 KB)—Figure S1: The workflow of the study design (DEOSGs: differentially expressed oxidative stress genes, DEGs: differentially expressed genes).

Additional file2 (TIF 12760 KB)—Figure S2: Identification of DEOSGs in CC from the TCGA database. (A) The heatmap showed the expression levels of the 50 genes that show the greatest difference in increase and decrease, respectively. Red represents high expression and green represents low expression. (B) The volcano plot showed 103 up-regulated genes (red) and 101 down-regulated genes (green) in the TCGA cohort (P < 0.05). Black dots mean meaningless (P > 0.05)

Additional file3 (TIF 1826 KB)—Figure S3: Univariate Cox regression analysis of DEOSGs in CC from the TCGA database. (A) Univariate Cox regression analysis for identification prognostic oxidative stress genes in CC (P < 0.05). HR > 1 are high-risk genes, indicated in red, and HR < 1 are low-risk genes, indicated in green. 95% confidence intervals for HR values were in brackets. (B) Waterfall chart of mutation frequency of 23 oxidative stress genes. (C) Multi-gene correlation map was generated using the 23 oxidative stress genes. A positive value represents a positive correlation; otherwise, a negative correlation. The larger correlation value means a better correlation between two genes. P < 0.01 is indicated with *

Additional file4 (TIF 7605 KB)—Figure S4: Construction and evaluation of oxidative stress-related risk signature in the TCGA cohort. (A) Cross-validation of the LASSO regression. (B) LASSO regression of the 16 prognostic oxidative stress genes. (C) PCA plot for oxidative stress (OS) genes. (D) PCA plot for 16 model genes. (E) Kaplan–Meier curves for comparison of the overall survival between low- and high-risk groups in the TCGA database (P < 0.001), (F) and the GSE17538 database (P = 0.010). (G) PFS analysis between low- and high-risk groups in the TCGA database (P < 0.001)

Additional file5 (TIF 11135 KB)—Figure S5: The independent prognostic value and clinicopathological feature of the risk score. (A) Univariate cox regression analysis for the TCGA cohort. (B) Multivariate cox regression analysis for the TCGA cohort. (C) Time ROC curves for forecasting overall survival in TCGA cohort. (D) Clinical ROC curves were used to evaluate the predictive accuracy of the risk score by forecasting overall survival in the TCGA cohort. (E) The relationship between the risk scores and T stage in TCGA cohort. (F) The relationship between the risk scores and N stage in the TCGA cohort. (G) The relationship between the risk scores and M stage in the TCGA cohort. (H) The relationship between the risk scores and tumor stage in the TCGA cohort

Additional file6 (TIF 8596 KB)—Figure S6: Construction and evaluation of oxidative stress-related nomogram in the TCGA cohort. (A) Nomogram of risk score and other clinical features for predicting CC 1-, 3-, and 5-year overall survival in TCGA cohort. (B) The calibration plot of the 1-year, 3-year, and 5-year survival rates of the nomogram in the TCGA cohort. (C) Clinical ROC curves were used to evaluate the predictive accuracy of the nomogram by forecasting overall survival in the TCGA cohort. (D) Univariate cox regression analysis for the nomo risk score in TCGA cohort. (E) Multivariate cox regression analysis for the nomo risk score in TCGA cohort

Additional file7 (TIF 9974 KB)—Figure S7: Differential analysis of immune cells and GSVA analysis. (A) CIBERSORT score of 22 immune cell infiltrations among TCGA samples of high- and low-risk groups. *p < 0.05; **p < 0.01; ***p < 0.001. (B) Comparison of proportions of different kinds of cells estimated by MCP-counter algorithm. *p < 0.05; **p < 0.01; ***p < 0.001. (C) GSVA heatmap of oxidative stress-related genes of high- and low-risk groups. Red represents high risk and the green represents low risk

Additional file8 (TIF 3592 KB)—Figure S8: Functional analysis of immune cells. (A) Bubble graph for GO enrichment in the TCGA cohort. BP: biological process; CC, cell component; MF, molecular function. (B) Circle diagram which enriched in GO analysis. (C) Barplot graph for KEGG pathways in the TCGA cohort. (D) Circle diagram which enriched in KEGG pathways

Additional file9 (TIF 6339 KB)—Figure S9: (A-B) Three other samples of immunohistochemical analysis of SERPINE1 expression in CC patients. *p < 0.05; **p < 0.01; ***p < 0.001.

Additional file10 (PDF 164 KB)—Figure S10: Full western blot images.

Additional file11 (XLS 10 KB)—Table S1: The detailed information of the patients was shown in Supplementary Table S1.

Additional file12 (CSV 58 KB)—Table S2: 433 oxidative stress protein domains with correlation scores ≥ 10 were extracted from the gene cards.

Additional file13 (XLSX 27 KB)—Table S3: The detailed information of DEOSGs in the TCGA cohort was shown in Supplementary Table S3.

Additional file14 (XLS 7 KB)—Table S4: The detailed information of DEGs in TCGA cohort was shown in Supplementary Table S4.

## Data Availability

The raw transcriptome data of this study are available in the TCGA database (TCGA cohorts) (https://portal.gdc.cancer.gov/) and the GEO database (GSE17538) (https://www.ncbi.nlm.nih.gov/gds/). Oxidative stress genes were downloaded and collated from Genecards (https://www.genecards.org/).

## Abbreviations

CC: Colon cancer
ROS: Reactive oxygen radicals
RNS: Reactive nitrogen radicals
TCGA: The Cancer Genome Atlas
GEO: Gene expression omnibus
FPKM: Fragments per kilobase of exon model per Million mapped fragments
DEOSGs: Differentially expressed oxidation stress genes
HR: Hazard ratio
PFS: Progression-free survival
ROC: Receiver operating characteristic curve
AUC: Area under the curve
MSigDB: Molecular signatures database
GSVA: Gene set variation analysis
DEGs: Differentially expressed genes
PPI: Protein–protein interaction
EPC: Edge percolated component
WB: Western blot
IHC: Immunohistochemistry
ROS: Reactive oxygen species
MDA: Malonic dialdehyde
GSH: Micro reduced glutathione
