# Supplementary figures and images for: Novel diagnostic biomarkers of oxidative stress, immune- infiltration characteristics and experimental validation of SERPINE1 in colon cancer
Source: Discov Oncol. 2023 Nov 18;14:206. doi: 10.1007/s12672-023-00833-w (PMC10657345; doi:10.1007/s12672-023-00833-w)

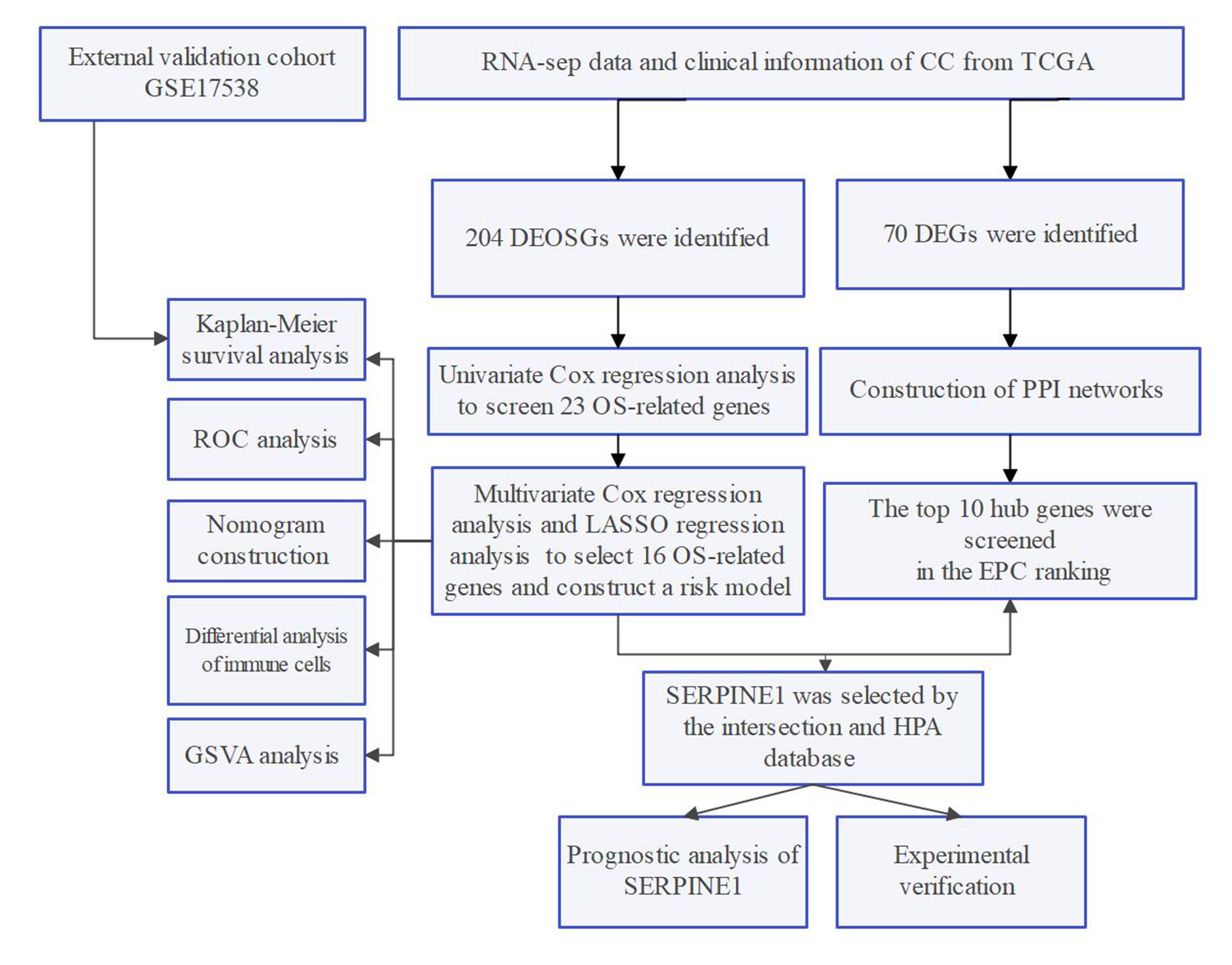

Supplement: Supplementary file 1 — Additional file1 (TIF 9283 KB)—Figure S1: The workflow of the study design (DEOSGs: differentially expressed oxidative stress genes, DEGs: differentially expressed genes). [file 12672_2023_833_MOESM1_ESM.tif]

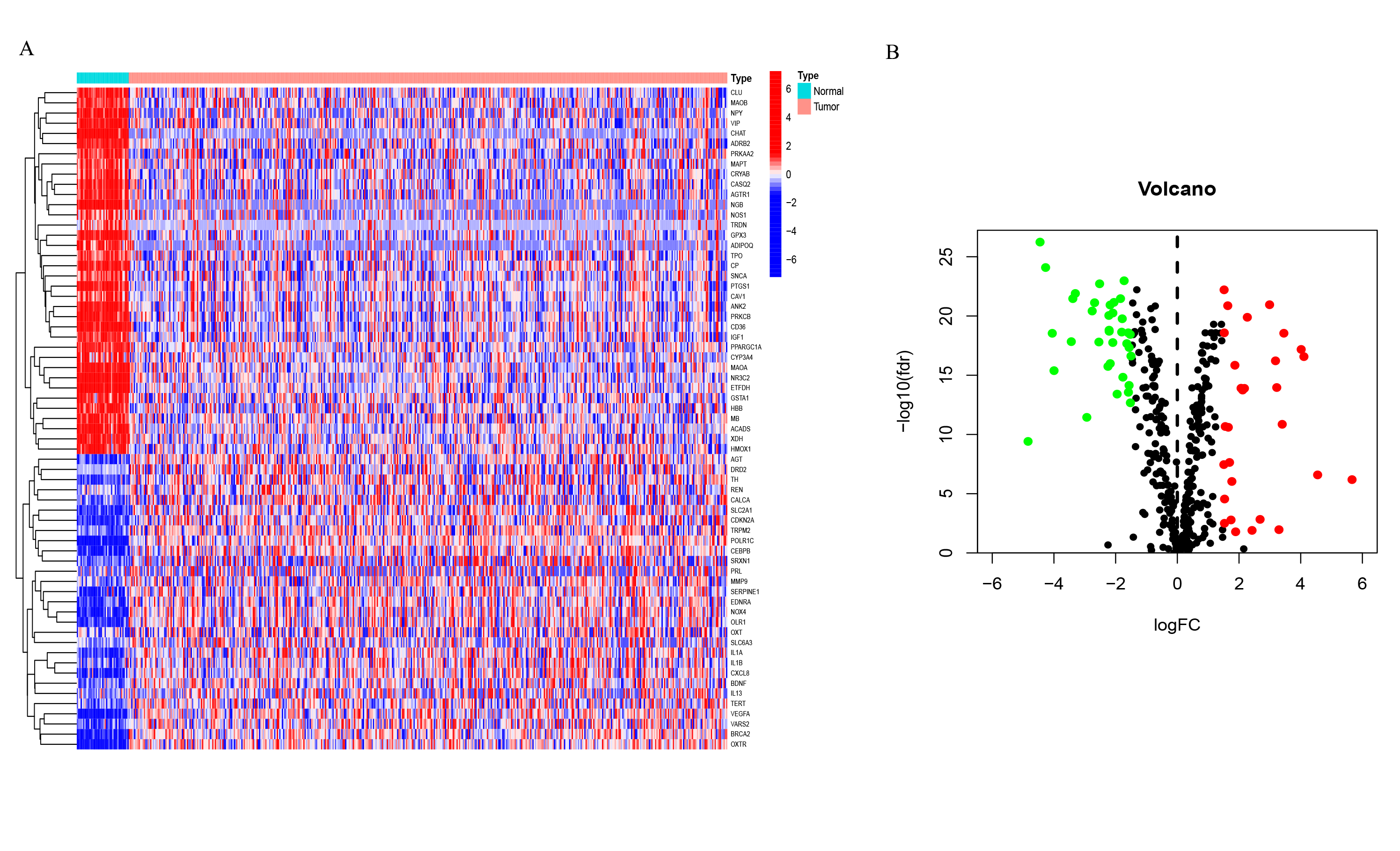

Supplement: Supplementary file 2 — Additional file2 (TIF 12760 KB)—Figure S2: Identification of DEOSGs in CC from the TCGA database. (A) The heatmap showed the expression levels of the 50 genes that show the greatest difference in increase and decrease, respectively. Red represents high expression and green represents low expression. (B) The volcano plot showed 103 up-regulated genes (red) and 101 down-regulated genes (green) in the TCGA cohort (P < 0.05). Black dots mean meaningless (P > 0.05) [file 12672_2023_833_MOESM2_ESM.tif]

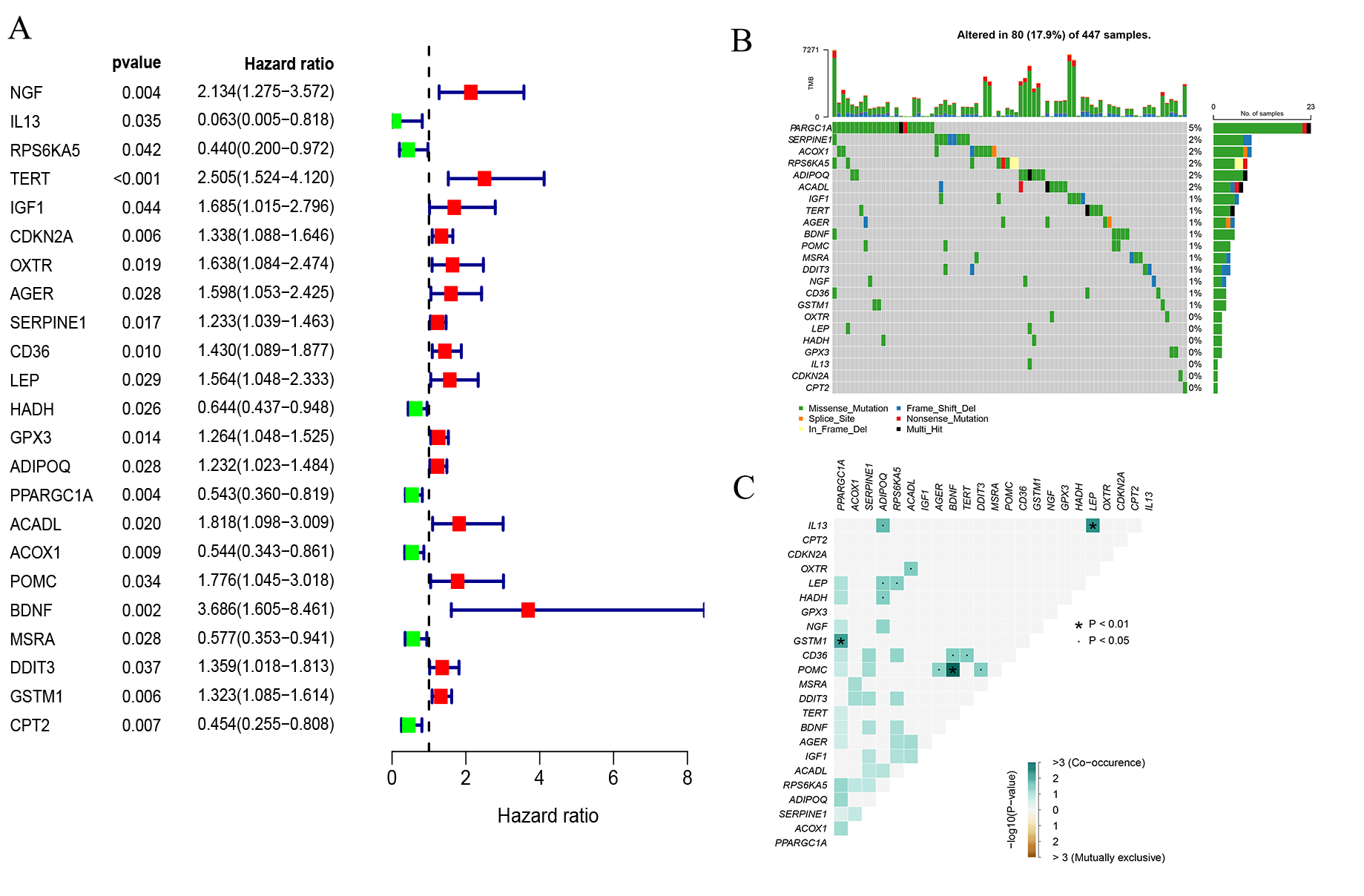

Supplement: Supplementary file 3 — Additional file3 (TIF 1826 KB)—Figure S3: Univariate Cox regression analysis of DEOSGs in CC from the TCGA database. (A) Univariate Cox regression analysis for identification prognostic oxidative stress genes in CC (P < 0.05). HR > 1 are high-risk genes, indicated in red, and HR < 1 are low-risk genes, indicated in green. 95% confidence intervals for HR values were in brackets. (B) Waterfall chart of mutation frequency of 23 oxidative stress genes. (C) Multi-gene correlation map was generated using the 23 oxidative stress genes. A positive value represents a positive correlation; otherwise, a negative correlation. The larger correlation value means a better correlation between two genes. P < 0.01 is indicated with * [file 12672_2023_833_MOESM3_ESM.tif]

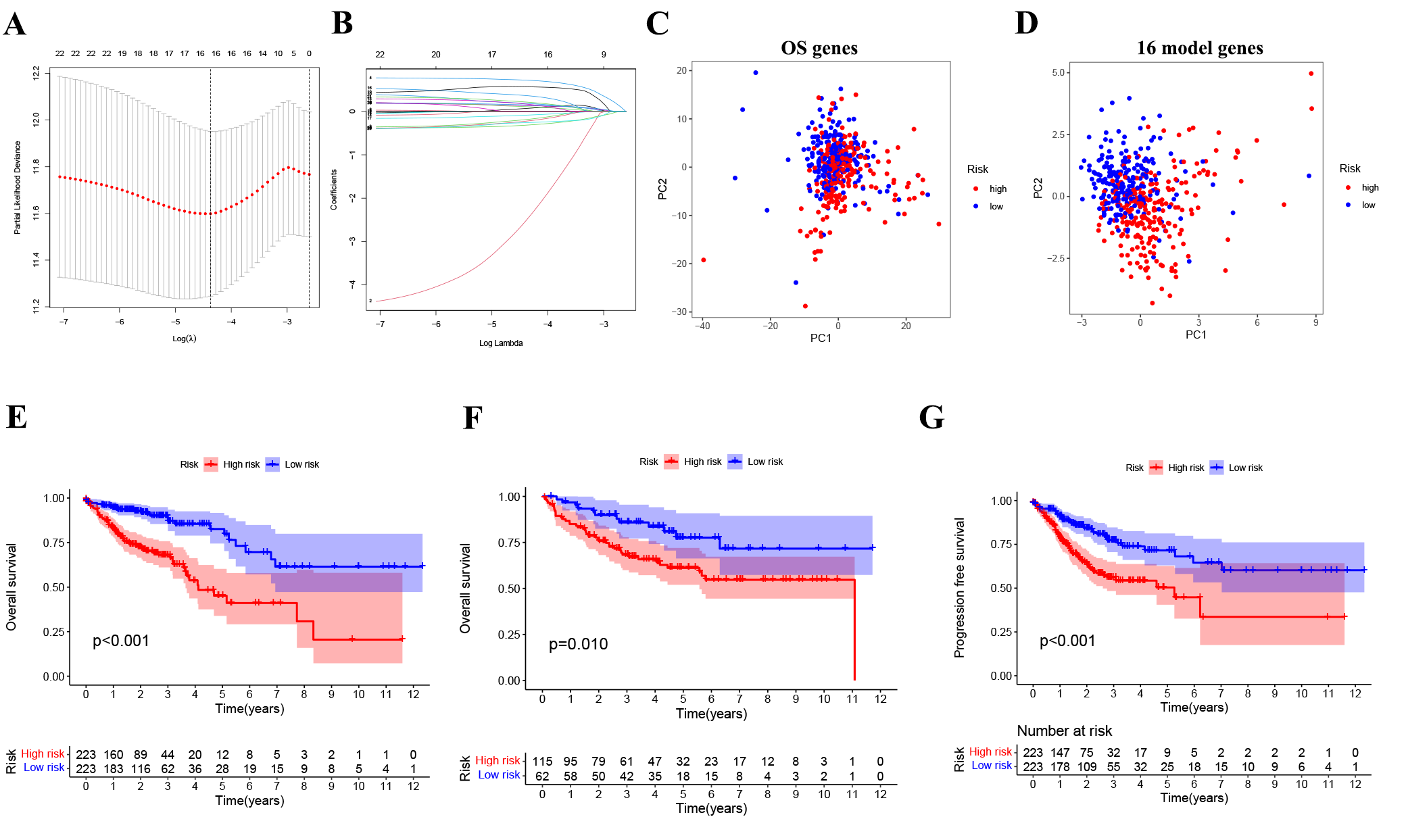

Supplement: Supplementary file 4 — Additional file4 (TIF 7605 KB)—Figure S4: Construction and evaluation of oxidative stress-related risk signature in the TCGA cohort. (A) Cross-validation of the LASSO regression. (B) LASSO regression of the 16 prognostic oxidative stress genes. (C) PCA plot for oxidative stress (OS) genes. (D) PCA plot for 16 model genes. (E) Kaplan–Meier curves for comparison of the overall survival between low- and high-risk groups in the TCGA database (P < 0.001), (F) and the GSE17538 database (P = 0.010). (G) PFS analysis between low- and high-risk groups in the TCGA database (P < 0.001) [file 12672_2023_833_MOESM4_ESM.tif]

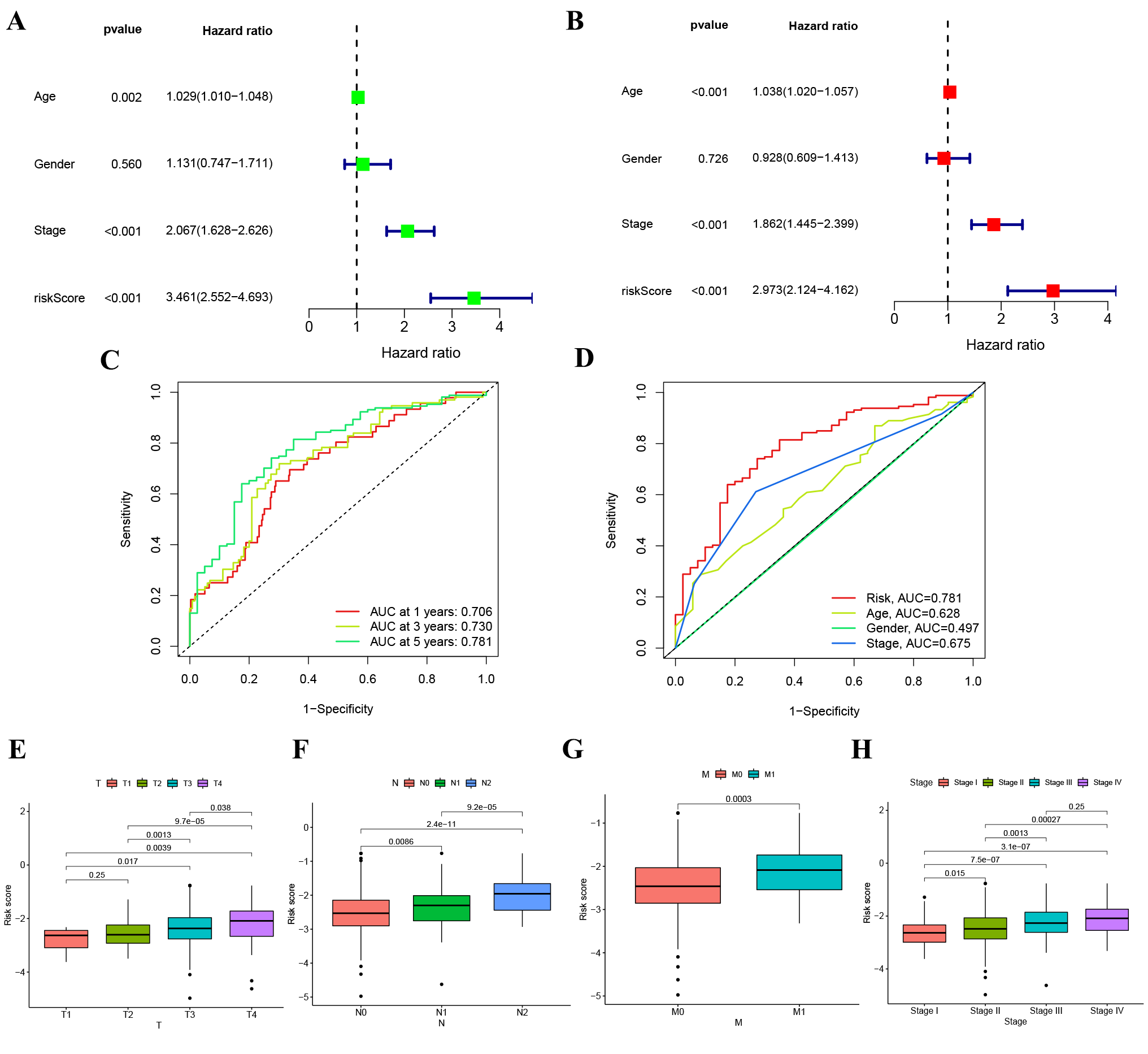

Supplement: Supplementary file 5 — Additional file5 (TIF 11135 KB)—Figure S5: The independent prognostic value and clinicopathological feature of the risk score. (A) Univariate cox regression analysis for the TCGA cohort. (B) Multivariate cox regression analysis for the TCGA cohort. (C) Time ROC curves for forecasting overall survival in TCGA cohort. (D) Clinical ROC curves were used to evaluate the predictive accuracy of the risk score by forecasting overall survival in the TCGA cohort. (E) The relationship between the risk scores and T stage in TCGA cohort. (F) The relationship between the risk scores and N stage in the TCGA cohort. (G) The relationship between the risk scores and M stage in the TCGA cohort. (H) The relationship between the risk scores and tumor stage in the TCGA cohort [file 12672_2023_833_MOESM5_ESM.tif]

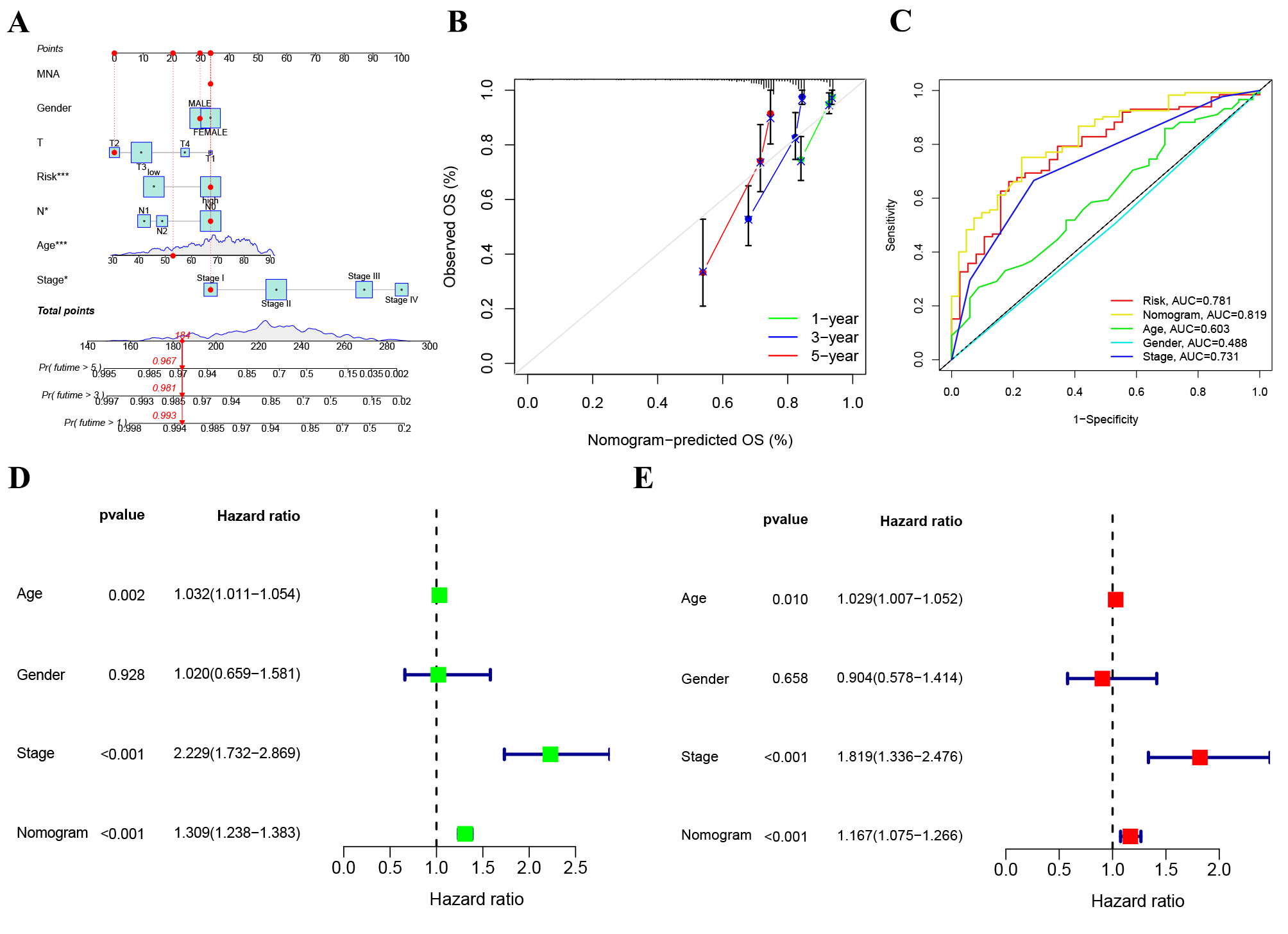

Supplement: Supplementary file 6 — Additional file6 (TIF 8596 KB)—Figure S6: Construction and evaluation of oxidative stress-related nomogram in the TCGA cohort. (A) Nomogram of risk score and other clinical features for predicting CC 1-, 3-, and 5-year overall survival in TCGA cohort. (B) The calibration plot of the 1-year, 3-year, and 5-year survival rates of the nomogram in the TCGA cohort. (C) Clinical ROC curves were used to evaluate the predictive accuracy of the nomogram by forecasting overall survival in the TCGA cohort. (D) Univariate cox regression analysis for the nomo risk score in TCGA cohort. (E) Multivariate cox regression analysis for the nomo risk score in TCGA cohort [file 12672_2023_833_MOESM6_ESM.tif]

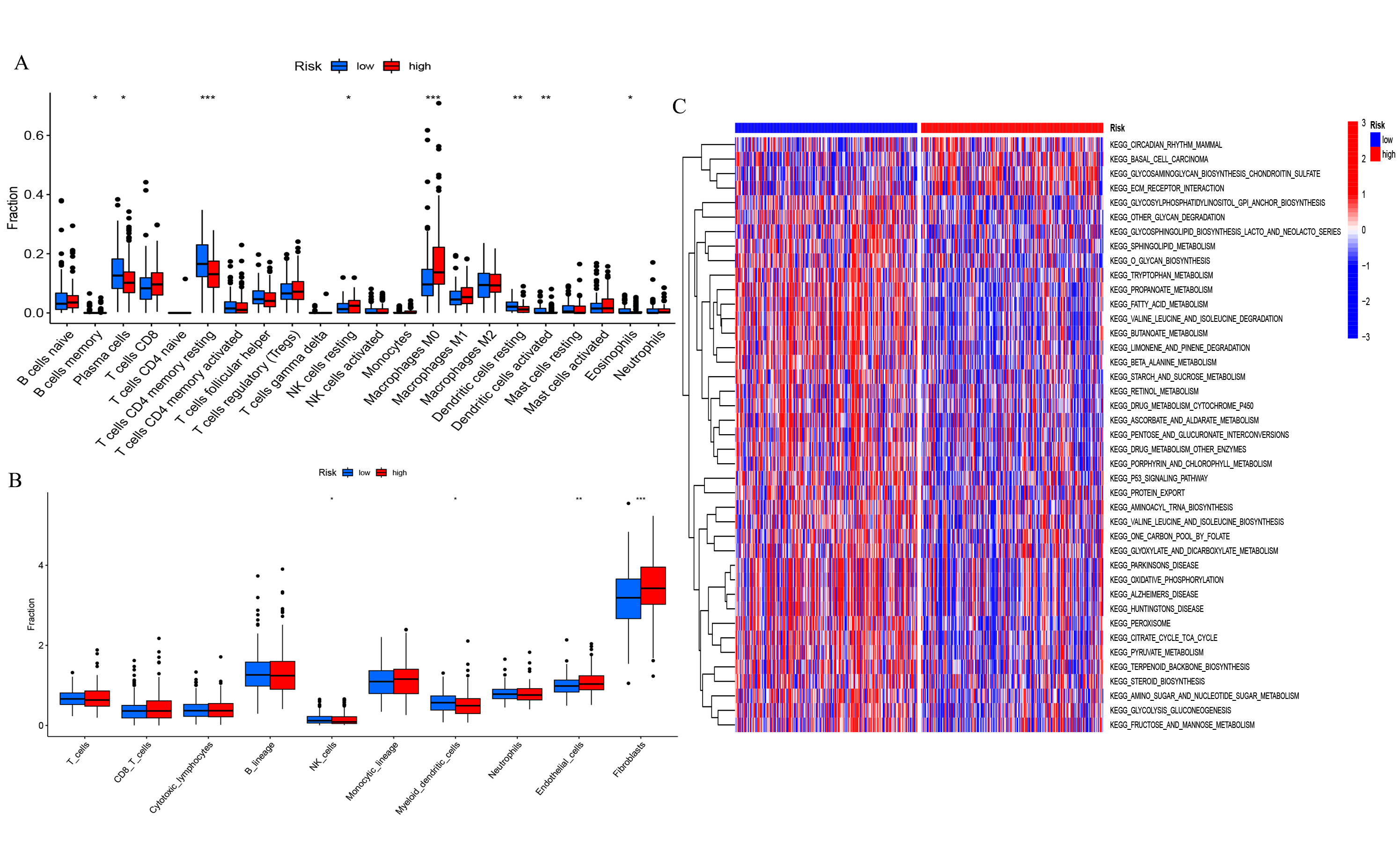

Supplement: Supplementary file 7 — Additional file7 (TIF 9974 KB)—Figure S7: Differential analysis of immune cells and GSVA analysis. (A) CIBERSORT score of 22 immune cell infiltrations among TCGA samples of high- and low-risk groups. *p < 0.05; **p < 0.01; ***p < 0.001. (B) Comparison of proportions of different kinds of cells estimated by MCP-counter algorithm. *p < 0.05; **p < 0.01; ***p < 0.001. (C) GSVA heatmap of oxidative stress-related genes of high- and low-risk groups. Red represents high risk and the green represents low risk [file 12672_2023_833_MOESM7_ESM.tif]

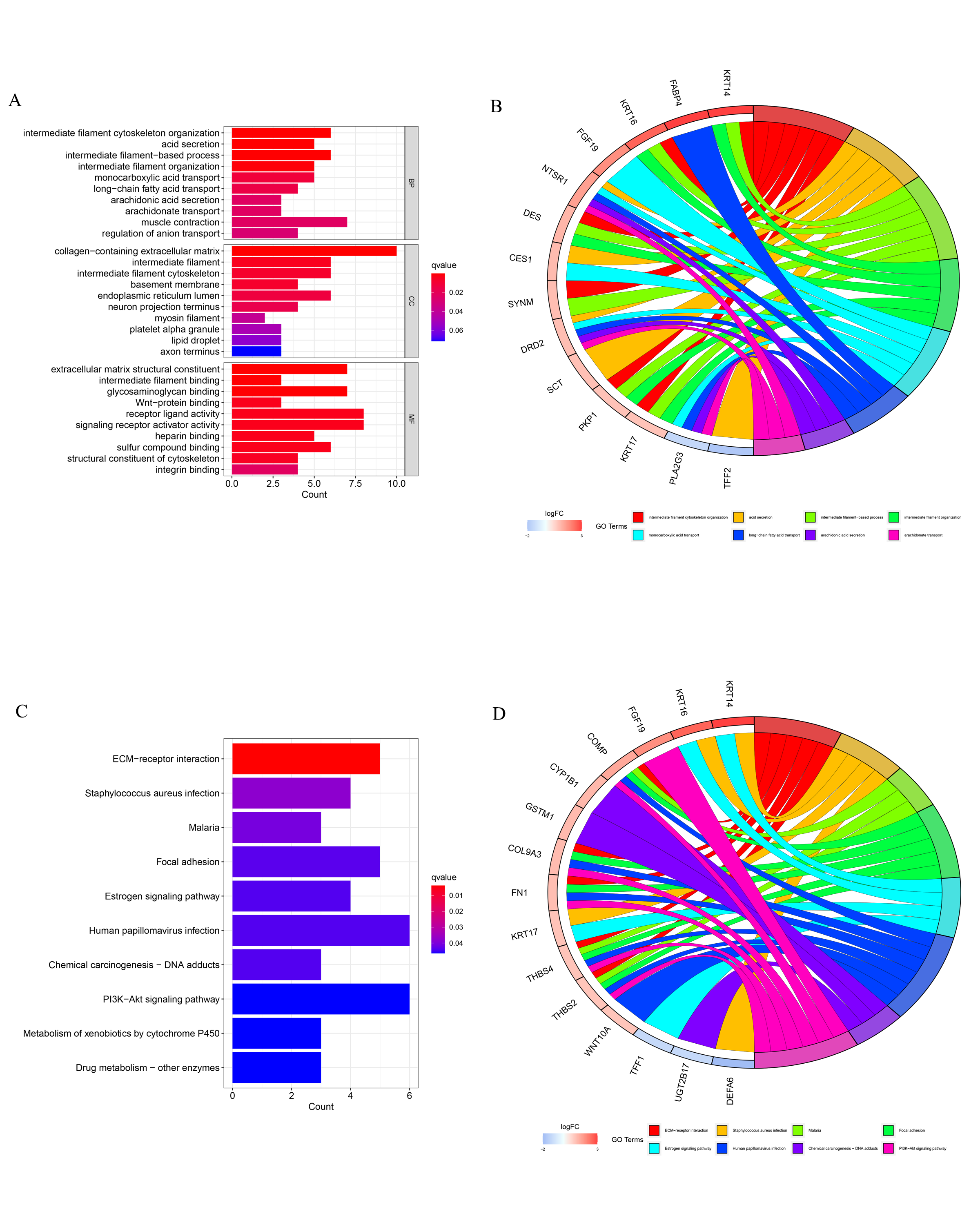

Supplement: Supplementary file 8 — Additional file8 (TIF 3592 KB)—Figure S8: Functional analysis of immune cells. (A) Bubble graph for GO enrichment in the TCGA cohort. BP: biological process; CC, cell component; MF, molecular function. (B) Circle diagram which enriched in GO analysis. (C) Barplot graph for KEGG pathways in the TCGA cohort. (D) Circle diagram which enriched in KEGG pathways [file 12672_2023_833_MOESM8_ESM.tif]

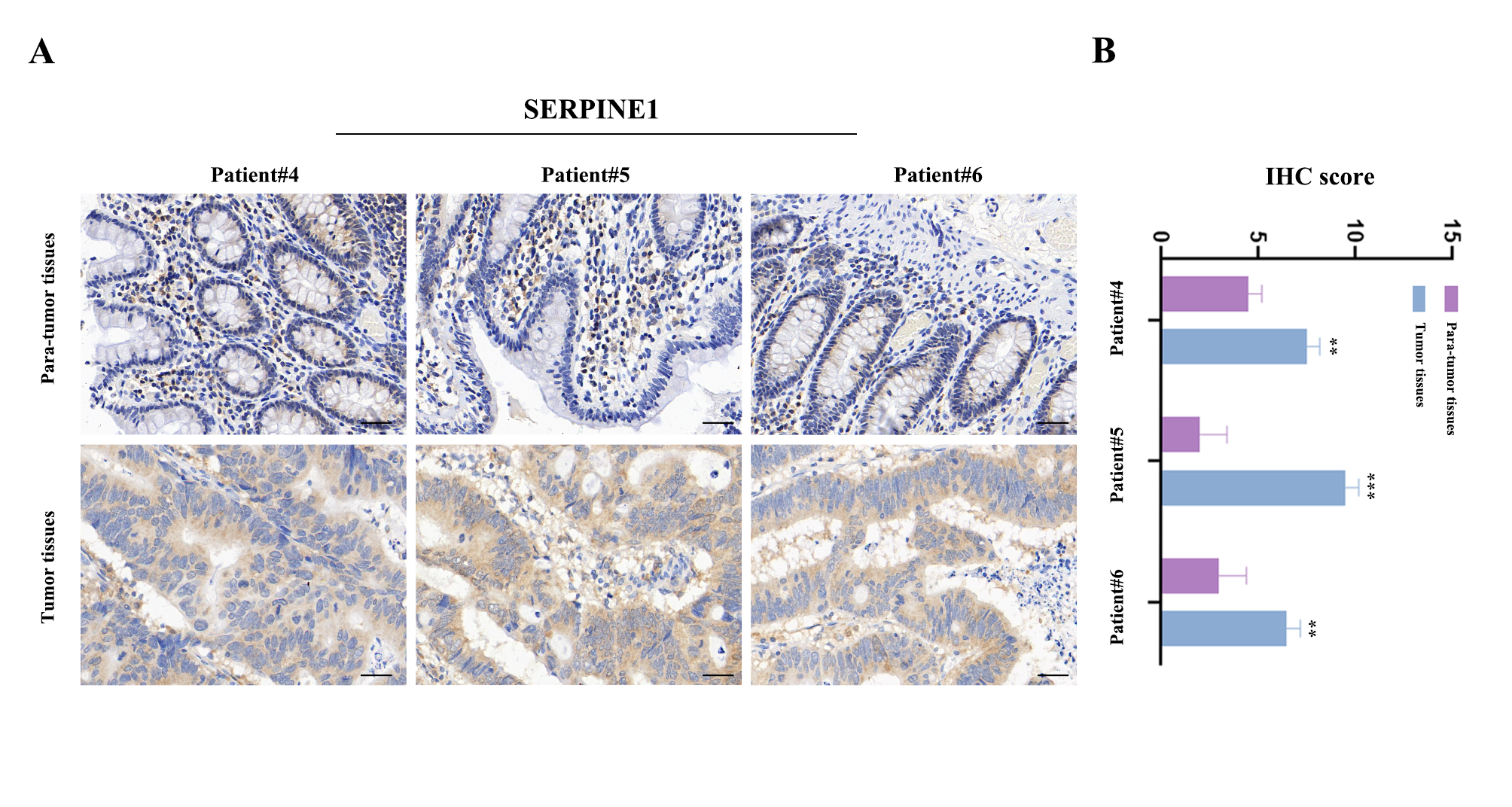

Supplement: Supplementary file 9 — Additional file9 (TIF 6339 KB)—Figure S9: (A-B) Three other samples of immunohistochemical analysis of SERPINE1 expression in CC patients. *p < 0.05; **p < 0.01; ***p < 0.001. [file 12672_2023_833_MOESM9_ESM.tif]

Figure 10B

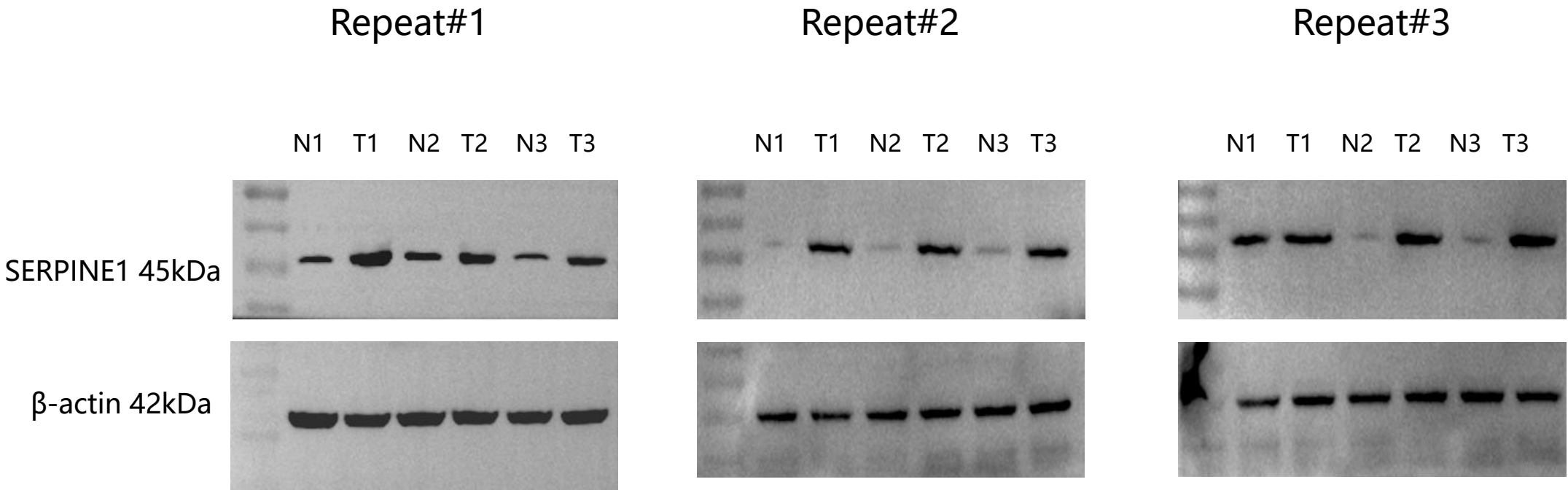

Figure 10B

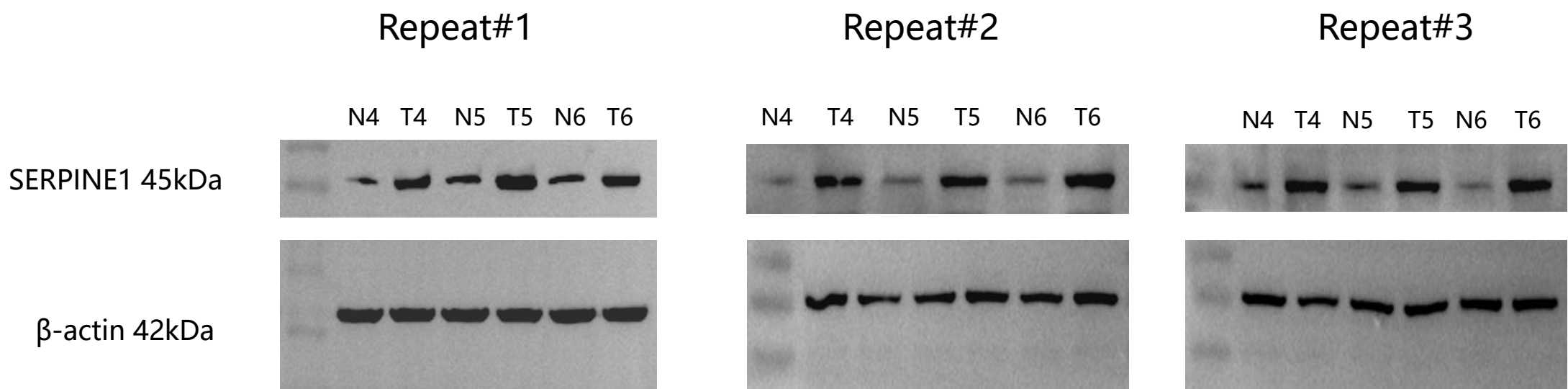

Figure 10D

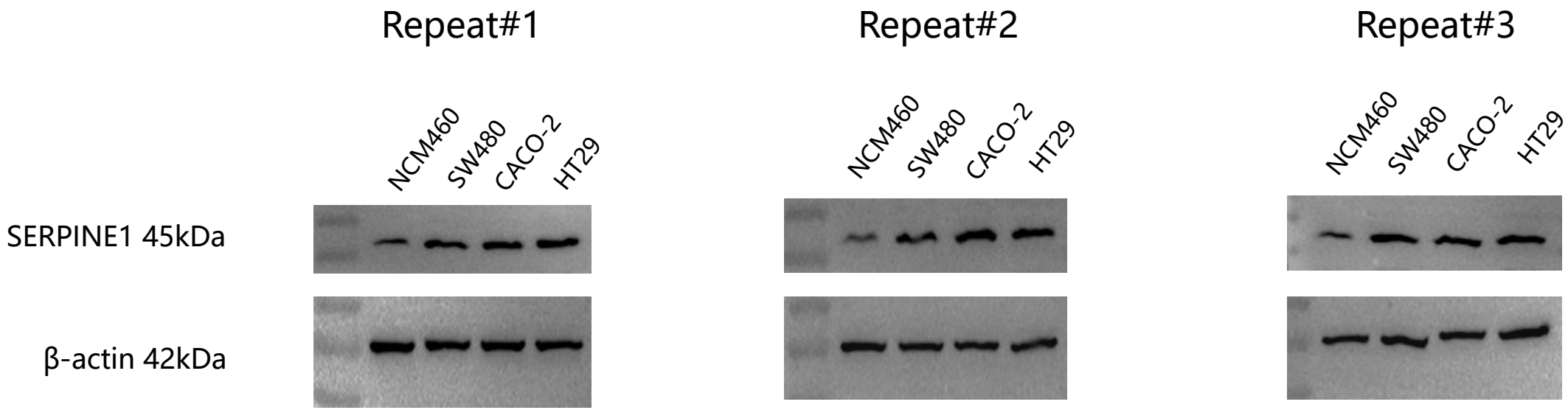

Figure 11A

Repeat#1

Repeat#2

Repeat#3

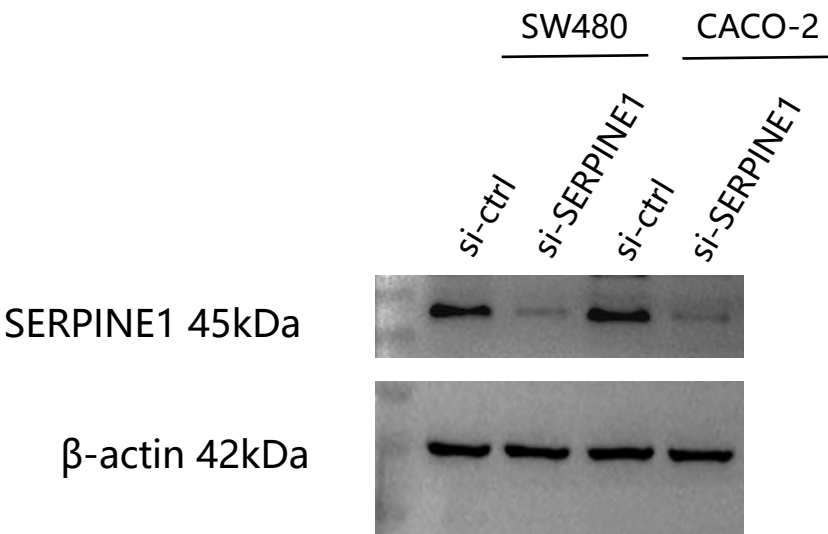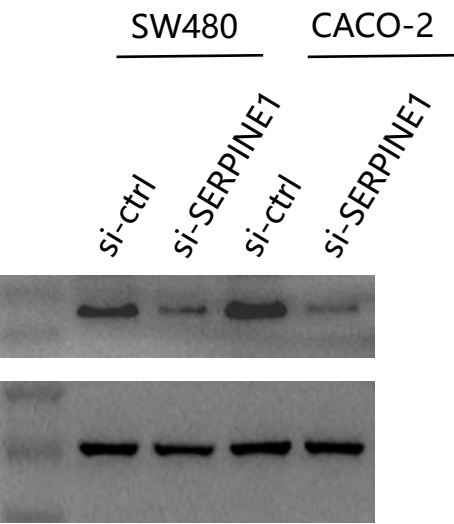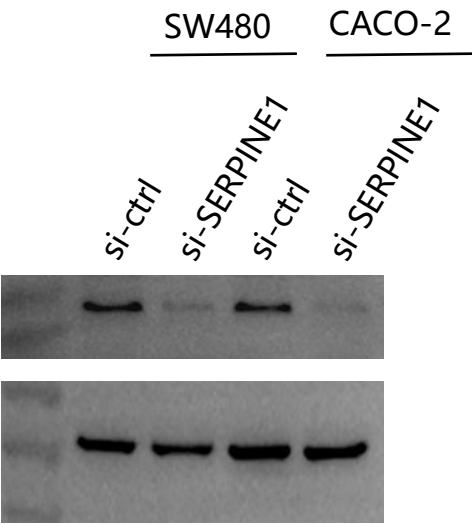

Supplement: Supplementary file 10 — Additional file10 (PDF 164 KB)—Figure S10: Full western blot images. [file 12672_2023_833_MOESM10_ESM.pdf]
